# Supplementary material for: Changes in the top 25 reasons for primary care visits during the COVID-19 pandemic in a high-COVID region of Canada
Source: PLoS One. 2021 Aug 12;16(8):e0255992. doi: 10.1371/journal.pone.0255992 (PMC8360367; doi:10.1371/journal.pone.0255992)
Supplement: S1 Appendix — (DOCX) [file pone.0255992.s001.docx]

**S1 Appendix: Inferred diagnoses for missing diagnostic codes**

| **OHIP Service Code** | **Service Code Description** | **Inferred Diagnostic code** | **Diagnostic code Description** |
| --- | --- | --- | --- |
| K030 | Diabetic management assessment | 250 | Diabetes Mellitus (including complications) |
| P003 | General assessment (major prenatal visit) | 650 | Normal delivery, uncomplicated pregnancy |
| P004 | Minor prenatal assessment |  |  |
| P005 | Antenatal preventative health assessment |  |  |
| P008 | Postnatal care in office |  |  |
| A002 | Enhanced 18 month well baby visit | 916 | Well baby care |
| K017 | Periodic health visit child | 917 | Annual health examination adolescent/ adult, Well Vision Care |
| K130 | Periodic health visit adolescent |  |  |
| K131 | Periodic health visit adult age 18 to 64 inclusive |  |  |
| K132 | Periodic health visit adult 65 years of age and older |  |  |

*Reference:*  Ontario Ministry of Health and Longterm Care. Resource Manual for Physicians. 2015. Section 4: Claims Submission.
