# Supplementary material for: Changes in the top 25 reasons for primary care visits during the COVID-19 pandemic in a high-COVID region of Canada
Source: PLoS One. 2021 Aug 12;16(8):e0255992. doi: 10.1371/journal.pone.0255992 (PMC8360367; doi:10.1371/journal.pone.0255992)
Supplement: S2 Appendix — (DOCX) [file pone.0255992.s002.docx]

**S2 Appendix: Data quality criteria**

Family physician data were eligible for inclusion if the data quality assessment criteria were met as follows:

1. The percent of rostered patients with a billing record in the year prior to the CutOffDate (Bills_ratio) is greater than or equal to 20%
2. The percent of rostered adult patients with a ‘selected’ lab test in the year prior to the CutOffDate (Lab_ratio) is greater than or equal to 20%
3. The percent of rostered adult patients with a medication record in the year prior to the CutOffDate (Medication_ratio) is greater than or equal to 20%
4. At least 200 rostered patients

Criteria taken from: Tu K, Sodhi S, Kidd M, et al. The University of Toronto Family Medicine Report: Caring for our Diverse Populations. 2020. Technical Appendix.

The **CutOffDate** for the data used in the current study was December 31, 2020.

**Rostered patients:** In Ontario, patient rostering is voluntary and can be used with specific renumeration models. All UTOPIAN contributing family physicians’ practice under a rostering model of care. Rostered patients commit to seek treatment from their enrolling physician or group to which the family physician belongs unless they are traveling or find themselves in an emergency situation. Physicians commit to provide comprehensive care to their rostered patients.
