# Supplementary material for: Changes in the top 25 reasons for primary care visits during the COVID-19 pandemic in a high-COVID region of Canada
Source: PLoS One. 2021 Aug 12;16(8):e0255992. doi: 10.1371/journal.pone.0255992 (PMC8360367; doi:10.1371/journal.pone.0255992)
Supplement: S3 Appendix — (DOCX) [file pone.0255992.s003.docx]

**S3 Appendix: Eligible Service Codes**

**Family practice visits**

OHIP service codes were used to select primary care visits that occurred via telephone, video, or in-person with family physician. A primary care visit was considered to have occurred on any date with any of the following service codes billed for an eligible patient.

| Code | Description | Code | Description |
| --- | --- | --- | --- |
| A001 | minor assessment | K017 | periodic health visit-child aft. 2nd birthday |
| A002 | enhanced 18-month well baby visit | K022 | hiv prim care individ care 1/2 hr or major part |
| A003 | major assessment | K028 | sexually transmitted disease (std) counseling |
| A004 | general re-assessment | K030 | diabetic management fee |
| A007 | intermediate assessment | K032 | gp-specific neurocognitive assessment |
| A008 | mini assessment | K033 | counselling - 1 pt/yr/unit |
| A071 | complex medical specific re-assessment | K039 | smoking cessation follow-up visit |
| A131 | complex medical specific re-assessment | K130 | periodic health visit - adolescent |
| A134 | medical specific re-assessment | K131 | periodic health visit - adult aged 18 to 64 inclusive |
| A624 | medical specific re-assessment | K132 | periodic health visit - adult 65 years of age and older |
| A888 | partial assessment | K680 | substance abuse - extended assessment |
| A903 | pre-op assessment | P003 | obs.-prenatal care-gen.assess-major prenatal visit |
| A920 | medical management of early pregnancy, initial visit | P004 | obs.-prenatal care-minor prenatal assess.-subseq.prenat.vis. |
| K005 | primary mental health | P005 | antenatal health screen |
| K007 | ind. psychotherapy per half hour - gp | P008 | obs.-post-natal care in office |
| K013 | counselling-one or more people-per 1/2hr | K037 | fibromyalgia/chronic fatigue syndrome care |
| K080 | Minor assessment of patient by telephone or video | K081 | Intermediate assessment including psychotherapy by telephone or video |
| K082 | Psychotherapy, psychiatric or mental health counselling by telephone or video | K087 | Minor assessment of an uninsured by telephone or video |
| K088 | Intermediate assessment of an uninsured patient including psychotherapy by telephone or video | K089 | Psychotherapy, psychiatric or mental health counselling of an uninsured patient by telephone or video |

**Visit format**

Two visit formats were defined: ***virtual*** (i.e., telephone or video) and ***in-person***.

Virtual visits were defined based on the use of virtual visit billing codes introduced specifically in response to the COVID-19 pandemic or based on the use of codes for billing services to the Ontario Telehealth Network (a virtual care service that was implemented prior to the COVID-19 pandemic).

A ***virtual visit*** was counted for any date on which any of the following conditions were met:

1. A service code for a family physician visit was billed (as defined above) and an applicable Virtual Care Program (OTN) service code was billed (B099, B101, B102, B103, B201, B202, B203).
2. Any of the OHIP codes for family physician visits by telephone or video were billed: K080, K081, K082, K087, K088, K089.

An ***in-person visit*** was counted for any date on which any of the following criteria were met:

1. A service code for a family physician visit was billed (as defined above excluding visits by telephone or video -- K080, K081, K082, K087, K088, K089) AND no Virtual Care Program (OTN) service code was billed (B099, B101, B102, B103, B201, B202, B203).
2. A Virtual Care Program (OTN) service code and 2 or more service codes for a family physician visit (as defined above excluding visits by telephone or video -- K080, K081, K082, K087, K088, K089) were billed on the same date.

Adapted from: Tu K, Sodhi S, Kidd M, et al. The University of Toronto Family Medicine Report: Caring for our Diverse Populations. 2020. Technical Appendix.
