# Supplementary material for: Changes in the top 25 reasons for primary care visits during the COVID-19 pandemic in a high-COVID region of Canada
Source: PLoS One. 2021 Aug 12;16(8):e0255992. doi: 10.1371/journal.pone.0255992 (PMC8360367; doi:10.1371/journal.pone.0255992)
Supplement: S4 Appendix — (DOCX) [file pone.0255992.s004.docx]

**S4 Appendix: Full diagnostic descriptions**

| OHIP Diagnostic code | OHIP Diagnostic code Description |
| --- | --- |
| 250 | diabetes mellitus (including complications) |
| 272 | Disorders of lipoid metabolism (e.g., hypercholesterolemia, lipoprotein disorders) |
| 300 | anxiety neurosis, hysteria, neurasthenia, obsessive compulsive neurosis, reactive depression, suicide tendencies |
| 311 | depressive or other non-psychotic disorders, not elsewhere classified |
| 401 | essential, benign hypertension |
| 460 | common cold, upper respiratory infection, acute nasopharyngitis, pharyngitis |
| 599 | hematuria, hemiplegia, other disorders of urinary tract |
| 650 | normal delivery, uncomplicated pregnancy |
| 691 | eczema, atopic dermatitis, neurodermatitis, rash |
| 709 | other disorders of skin and subcutaneous tissue, hirsutism, scar, scarring |
| 715 | osteoarthritis, non-specified chronic arthritis |
| 724 | lumbar strain, lumbago, coccydynia, sciatica, back pain |
| 727 | synovitis, tenosynovitis, bursitis, bunion, ganglion, Baker's cyst |
| 780 | signs and symptoms not yet diagnosed: convulsions, ataxia, vertigo, headache, except tension headache and migraine |
| 781 | signs and symptoms not yet diagnosed: leg cramps, leg pain, muscle pain, joint pain, arthralgia, joint swelling, masses |
| 785 | signs and symptoms not yet diagnosed: chest pain, tachycardia, syncope, shock, edema, masses |
| 786 | signs and symptoms not yet diagnosed: epistaxis, hemoptysis, cough, dyspnea, masses, shortness of breath, hyperventilation, sleep apnea |
| 787 | signs and symptoms not yet diagnosed: anorexia, nausea and vomiting, heartburn, dysphagia, hiccough, hematemesis, jaundice, ascites, abdominal pain, melena, masses |
| 796 | other non-specific abnormal findings |
| 799 | asphyxia, excessive sweating, general symptoms, other ill-defined conditions |
| 847 | sprains, strains and other trauma: neck, low back, coccyx, torticollis, whiplash |
| 895 | family planning, contraceptive advice, advice on sterilization or abortion |
| 896 | immunization - all types |
| 916 | well baby care |
| 917 | annual health examination adolescent/adult, well vision care |

*Reference:*  Ontario Ministry of Health and Longterm Care. Resource Manual for Physicians. 2015. Section 4: Claims Submission.
