# Supplementary material for: Changes in the top 25 reasons for primary care visits during the COVID-19 pandemic in a high-COVID region of Canada
Source: PLoS One. 2021 Aug 12;16(8):e0255992. doi: 10.1371/journal.pone.0255992 (PMC8360367; doi:10.1371/journal.pone.0255992)
Supplement: Attachment — Submitted filename: Shifts in office and virtual primary care during the early COVID pandemic (CMAJ Feb 2021).pdf [file pone.0255992.s005.pdf]

Shifts in office and virtual primary care during the early COVID-19 pandemic in Ontario, Canada

Richard H. Glazier MD MPH, Michael E. Green MD MPH, Fangyun C. Wu MSc MAcc, Eliot Frymire MA, Alexander Kopp BA, Tara Kiran MD MSc

Cite as: *CMAJ* 2021 February 8;193:E200-10. doi: 10.1503/cmaj.202303

ABSTRACT

BACKGROUND: Globally, primary care changed dramatically as a result of the coronavirus disease 2019 (COVID-19) pandemic. We aimed to understand the degree to which office and virtual primary care changed, and for which patients and physicians, during the initial months of the pandemic in Ontario, Canada.

METHODS: This population-based study compared comprehensive, linked primary care physician billing data from Jan. 1 to July 28, 2020, with the same period in 2019. We identified Ontario residents with at least 1 office or virtual (telephone or video) visit during the study period. We compared trends in total physician visits, office visits and virtual visits before COVID-19 with trends after pandemic-related public health measures changed the delivery of care, according to various patient and physician characteristics. We used interrupted time series analysis to compare trends in the early and later halves of the COVID-19 period.

RESULTS: Compared with 2019, total primary care visits between March and July 2020 decreased by 28.0%, from 7.66 to 5.51 per 1000 people/day. The smallest declines were among patients with the highest expected health care use (8.3%), those who could not be attributed to a primary care physician (10.2%), and older adults (19.1%). In contrast, total visits in rural areas increased by 6.4%. Office visits declined by 79.1% and virtual care increased 56-fold, comprising 71.1% of primary care physician visits. The lowest uptake of virtual care was among children (57.6%), rural residents (60.6%) and physicians with panels of ≥ 2500 patients (66.0%).

INTERPRETATION: Primary care in Ontario saw large shifts from office to virtual care over the first 4 months of the COVID-19 pandemic. Total visits declined least among those with higher health care needs. The determinants and consequences of these major shifts in care require further study.

Primary care is considered the cornerstone of most health systems worldwide, and in higher-income countries, primary care visits are about 30 times more frequent than hospital admissions.[1] Health systems with greater availability of primary care are associated with increased access to care, reduced health inequities, better outcomes and lower costs.[2] Despite the centrality of primary care to health care systems, little is known about how it has been affected by coronavirus disease 19 (COVID-19).

On Mar. 11, 2020, the World Health Organization declared COVID-19 a global pandemic.[3] On Mar. 15, Ontario's Chief Medical Officer of Health issued a directive to ramp down elective surgeries and other nonemergent health services, and on Mar. 19, health care providers and organizations were directed to stop or substantially reduce all nonessential or elective services until further notice.[4] The Ontario Ministry of Health and the Ontario Medical Association negotiated the addition of temporary billing codes in the province's schedule of benefits to facilitate virtual care, effective as of Mar. 14 (Appendix 1, available at www.cmaj.ca/lookup/doi/10.1503/cmaj.202303/tab-related-content). In mid to late May 2020, the province undertook a phased resumption of certain in-person health professional services and surgeries.[4]

Initial reports from ongoing COVID-19-related surveys of primary care providers in Canada and the United States showed major disruptions to care, decreased payments, challenges keeping offices functioning, lack of personal protective equipment and widespread uptake of virtual care.[5,6] The degree to which virtual care — such as phone calls, video visits and secure text messages — replaced in-person office visits is not known. It is also not known which patients and physicians were most affected by the challenges to office-based practice or the change to virtual visits. We aimed to understand the degree to which office and virtual primary care changed, and for which patients and physicians, during the initial months of the COVID-19 pandemic in Ontario, Canada.

 © 2021 Joule Inc. or its licensors

Methods

Study design and setting

We conducted a retrospective, population-based analysis using linked health administrative data to assess changes in total visit volume and visit type in primary care for all residents in Ontario, from the pre-pandemic period to the period during which COVID-19-related restrictions on in-person consultations came into effect in mid-March 2020. We compared trends for the months of January through July for 2019 and 2020 to account for seasonality. Ontario is Canada's most populous province, with a 2020 population of 14 745 040 people.[7] All permanent residents have full coverage for necessary physician and hospital services, including primary care visits, without copayments or deductibles.

Data sources and collection

We used the following administrative databases: the Ontario Health Insurance Plan (OHIP) database for physician claims; the Registered Persons Database, which is Ontario's health care registry for eligible patients; Client Agency Provider Enrolment tables for patients in primary care enrolment models; the Corporate Provider Database for physicians in patient enrolment models; and the ICES Physician Database for physician characteristics. These data sets were linked using unique encoded identifiers and analyzed at ICES. ICES is an independent, nonprofit research institute whose legal status under Ontario's health information privacy law allows it to collect and analyze deidentified health care and demographic data, without consent, for health system evaluation and improvement.

Study population

For this study, primary care physicians were defined as family doctors and general practitioners, but not pediatricians or general internists. We included only comprehensive primary care physicians, defined as those meeting minimum visit levels, billing mainly primary care codes and providing a diversity of core primary care services.[8] Office and home visits were defined using relevant billing codes. Virtual care was defined as any primary care physician billing for telephone or video visits using either the temporary new virtual care codes or existing telemedicine codes. The temporary codes did not distinguish telephone from video visits and did not include asynchronous care such as email or text. Throughout both the 2019 and 2020 study periods, the Ontario Telemedicine Network supported video visits in secure, physical host sites. As of Nov. 15, 2019, video visits with patients in their home or other locations were also supported. From Apr. 1, 2020, onward, specific codes for virtual care using video through the Ontario Telemedicine Network were introduced, at the usual rate for office care (Appendix 1). Office, home and virtual visits were limited to 1 per patient per physician per day.

Patient and physician characteristics

We collected data on the following patient characteristics: age, sex, neighbourhood income, first-time registration for health care coverage, rurality, primary care enrolment model and expected health care use. We collected information on age and sex from the health care registry. We derived neighbourhood income using postal codes and the 2016 Canadian Census, divided into quintiles of equal size, with quintile 1 having the lowest income and quintile 5 the highest. We used first-time registration for health care coverage within the previous 10 years as a proxy for recent immigration, as most recent registrants are immigrants. We determined rurality using the Rurality Index of Ontario (RIO),[9] including large urban (RIO score of 0), urban (RIO score 1–9), small urban (RIO score 10–39) and rural (RIO score ≥ 40). Primary care enrolment models included enhanced fee-for-service, blended capitation, blended capitation with an interprofessional Family Health Team, not in a patient enrolment model (fee-for-service only) and not having sufficient visits to be attributable to a primary care physician.[10] Expected health care use was assessed using the Johns Hopkins Adjusted Clinical Groups Resource Utilization Bands, with 0 being no health care use and 5 being the highest expected use.[11] We also collected data on the following characteristics of primary care physicians: sex, age, country of graduation and panel size, which we derived from provincial and ICES physician databases. We analyzed visit rates and type of visit by these various patient and physician characteristics.

Statistical analysis

We calculated weekly counts of total, office, home and virtual visits from January to July of 2019 and 2020, focusing on visit counts in the COVID-19 period (Mar. 11 to July 28, 2020) and the pre-COVID-19 comparison period (Mar. 12 to July 29, 2019). We calculated mean daily rates of visits per thousand people in the COVID-19 period compared with the pre-COVID-19 period. The denominator for rates included all Ontario residents registered with the provincial health insurance plan in each period.

We used 2-sample z tests to compare visits in Mar. 11 to July 28, 2020 with those in 2019 for the same time period. Because major changes in visits were still occurring between Mar. 11 and Mar. 31, 2020, we conducted interrupted time series analyses from Apr. 1 to July 28, 2020, divided into roughly equal earlier (Apr. 1 to May 26, 2020) and later (May 27 to July 28, 2020) COVID-19 time periods. We calculated average daily physician visits in each week, accounting for long weekends. We did not detect any significant autocorrelation using the Durbin–Watson test in any outcome, so we performed ordinary least squares interrupted time series regression. We stratified all analyses by sex; sex-specific data are not reported because no major differences were found, but are available on request.

Ethics approval

The use of data in this project was authorized under section 45 of Ontario's *Personal Health Information Protection Act*, which does not require review by a Research Ethics Board.

Results

Table 1 summarizes the characteristics of all Ontario residents eligible for health care during the 2020 COVID-19 period (n = 14 574 884) and the 2019 pre-COVID-19 comparison period (n = 14 388 566), and the number of primary care visits by Ontarians according to visit location. The characteristics of the primary

Table 1 (part 1 of 2): Ontario population and primary care visits, Mar. 12 to July 29, 2019 (pre-COVID-19) and Mar. 11 to July 28, 2020 (COVID-19), by visit location and patient characteristics

Variable	No. (%) of population		No. (%) of office visits		No. (%) of home visits	
	Pre-COVID-19 *n* = 14 388 566	COVID-19 *n* = 14 574 884	Pre-COVID-19 *n* = 15 174 126	COVID-19 *n* = 3 206 492	Pre-COVID-19 *n* = 108 679	COVID-19 *n* = 43 568
Sex						
Female	7 330 105 (50.9)	7 408 475 (50.8)	8 810 115 (58.1)	1 812 564 (56.5)	67 082 (61.7)	26 237 (60.2)
Male	7 058 461 (49.1)	7 166 409 (49.2)	6 364 011 (41.9)	1 393 928 (43.5)	41 597 (38.3)	17 331 (39.8)
Age, yr						
≤ 18	2 918 996 (20.3)	2 965 429 (20.4)	2 065 021 (13.6)	445 611 (13.9)	8 610 (7.9)	635 (1.5)
19–29	2 024 156 (14.1)	2 059 474 (14.1)	1 763 122 (11.6)	382 921 (11.9)	2 376 (2.2)	438 (1.0)
30–44	2 903 298 (20.2)	2 958 978 (20.3)	2 832 859 (18.7)	601 487 (18.8)	5 441 (5.0)	885 (2.0)
45–64	4 011 605 (27.9)	4 011 526 (27.5)	4 662 559 (30.7)	961 289 (30.0)	13 642 (12.6)	4 725 (10.8)
65–74	1 431 818 (10.0)	1 460 363 (10.0)	2 079 437 (13.7)	427 604 (13.3)	13 943 (12.8)	6 895 (15.8)
≥ 75	1 098 693 (7.6)	1 119 114 (7.7)	1 771 128 (11.7)	387 580 (12.1)	64 667 (59.5)	29 990 (68.8)
Neighbourhood income quintile						
Q1 (lowest)	2 827 594 (19.6)	2 853 828 (19.6)	3 030 386 (20.0)	689 544 (21.5)	25 001 (23.0)	10 996 (25.2)
Q2	2 823 987 (19.6)	2 856 893 (19.6)	3 074 225 (20.3)	642 579 (20.0)	24 477 (22.5)	9 700 (22.3)
Q3	2 897 691 (20.1)	2 941 890 (20.2)	3 154 409 (20.8)	632 224 (19.7)	20 579 (18.9)	8 314 (19.1)
Q4	2 902 771 (20.2)	2 950 876 (20.2)	3 037 814 (20.0)	580 249 (18.1)	18 583 (17.1)	7 143 (16.4)
Q5 (highest)	2 912 107 (20.2)	2 946 225 (20.2)	2 833 515 (18.7)	511 030 (15.9)	19 646 (18.1)	7 196 (16.5)
Missing	24 416 (0.2)	25 172 (0.2)	43 777 (0.3)	150 866 (4.7)	393 (0.4)	219 (0.5)
Recent registrant < 10 yr						
No	11 487 764 (79.8)	11 511 755 (79.0)	12 280 000 (81.0)	2 508 772 (78.2)	97 551 (89.8)	42 044 (96.5)
Yes	1 259 553 (8.8)	1 381 008 (9.5)	1 437 718 (9.5)	321 240 (10.0)	2 847 (2.6)	882 (2.0)
Missing	1 641 249 (11.4)	1 682 121 (11.5)	1 452 134 (9.6)	376 480 (11.7)	8 281 (7.2)	642 (1.5)
Model of primary care						
Capitation	4 242 767 (29.5)	4 280 269 (29.4)	4 336 071 (28.6)	782 564 (24.4)	28 709 (26.4)	12 985 (29.8)
Enhanced fee-for-service	4 396 397 (30.6)	4 412 620 (30.3)	6 675 802 (44.0)	1 504 264 (46.9)	43 203 (39.8)	16 599 (38.1)
Family Health Team	3 579 169 (24.9)	3 600 281 (24.7)	2 745 367 (18.1)	451 139 (14.1)	21 809 (20.1)	9 355 (21.5)
Not in a patient enrolment model	1 034 343 (7.2)	1 036 289 (7.1)	950 271 (6.3)	234 302 (7.3)	11 160 (10.3)	3 554 (8.2)
Not attributable to a primary care physician	1 135 890 (7.9)	1 245 425 (8.6)	143 079 (0.9)	54 614 (1.7)	2 190 (2.0)	773 (1.8)
Rurality						
Large urban	6 080 663 (42.3)	6 186 490 (42.4)	6 869 774 (45.3)	1 418 637 (44.2)	49 744 (45.8)	21 738 (49.9)
Urban	4 363 444 (30.3)	4 428 280 (30.4)	5 377 869 (35.4)	1 060 515 (33.1)	32 780 (30.2)	9 708 (22.3)
Small urban	2 771 108 (19.3)	2 789 421 (19.1)	2 275 457 (15.0)	449 117 (14.0)	17 767 (16.4)	8 611 (19.8)
Rural	1 173 351 (8.2)	1 170 693 (8.0)	651 026 (4.3)	278 223 (8.7)	8 388 (7.7)	3 511 (8.1)
Johns Hopkins resource utilization bands						
0 (lowest user)	1 520 354 (10.6)	1 599 109 (11.0)	224 140 (1.5)	41 322 (1.3)	279 (0.3)	105 (0.2)
1	892 667 (6.2)	895 682 (6.2)	360 404 (2.4)	57 393 (1.8)	592 (0.5)	105 (0.2)
2	2 884 992 (20.0)	2 902 616 (19.9)	1 719 758 (11.3)	290 697 (9.1)	3 541 (3.3)	660 (1.5)
3	6 621 471 (46.0)	6 685 259 (45.9)	8 094 397 (53.3)	1 656 752 (51.7)	29 197 (26.9)	9 254 (21.2)
4	1 803 504 (12.5)	1 818 207 (12.5)	3 325 038 (21.9)	806 116 (25.1)	25 460 (23.4)	10 216 (23.4)
5 (highest user)	665 578 (4.6)	674 011 (4.6)	1 450 389 (9.6)	354 212 (11.0)	49 610 (45.6)	23 228 (53.3)

Table 1 (part 2 of 2): Ontario population and primary care visits, Mar. 12 to July 29, 2019 (pre-COVID-19) and Mar. 11 to July 28, 2020 (COVID-19), by visit location and patient characteristics

Variable	No. (%) of virtual visits		No. (%) of overall visits	
	Pre-COVID-19 *n* = 138 201	COVID-19 *n* = 7 991 197	Pre-COVID-19 *n* = 15 421 006	COVID-19 *n* = 11 241 257
Sex				
Female	79 351 (57.4)	4 830 485 (60.4)	8 956 548 (58.1)	6 669 286 (59.3)
Male	58 850 (42.6)	3 160 712 (39.6)	6 464 458 (41.9)	4 571 971 (40.7)
Age, yr				
≤ 18	18 908 (13.7)	605 839 (7.6)	2 092 539 (13.6)	1 052 085 (9.4)
19–29	26 663 (19.3)	893 940 (11.2)	1 792 161 (11.6)	1 277 299 (11.4)
30–44	32 229 (23.3)	1 548 393 (19.4)	2 870 529 (18.6)	2 150 765 (19.1)
45–64	39 574 (28.6)	2 639 766 (33.0)	4 715 775 (30.6)	3 605 780 (32.1)
65–74	12 899 (9.3)	1 200 650 (15.0)	2 106 279 (13.7)	1 635 149 (14.6)
≥ 75	7 928 (5.7)	1 102 609 (13.8)	1 843 723 (12.0)	1 520 179 (13.5)
Neighbourhood income quintile				
Q1 (lowest)	32 909 (23.8)	1 556 239 (19.5)	3 088 296 (20.0)	2 256 779 (20.1)
Q2	28 516 (20.6)	1 605 729 (20.1)	3 127 218 (20.3)	2 258 008 (20.1)
Q3	26 409 (19.1)	1 638 368 (20.5)	3 201 397 (20.8)	2 278 906 (20.3)
Q4	27 237 (19.7)	1 572 847 (19.7)	3 083 634 (20.0)	2 160 239 (19.2)
Q5 (highest)	22 730 (16.4)	1 501 010 (18.8)	2 875 891 (18.7)	2 019 236 (18.0)
Missing	400 (0.3)	117 004 (1.5)	44 570 (0.3)	268 089 (2.4)
Recent registrant < 10 yr				
No	113 795 (82.3)	6 820 926 (85.4)	12 491 346 (81.0)	9 371 742 (83.4)
Yes	10 977 (7.9)	737 944 (9.2)	1 451 542 (9.4)	1 060 066 (9.4)
Missing	13 429 (9.7)	432 327 (5.4)	1 473 844 (9.6)	809 449 (7.2)
Model of primary care				
Capitation	32 022 (23.2)	2 179 016 (27.3)	4 396 802 (28.5)	2 974 565 (26.5)
Enhanced fee-for-service	49 370 (35.7)	3 709 847 (46.4)	6 768 375 (43.9)	5 230 710 (46.5)
Family Health Team	32 228 (23.3)	1 437 610 (18.0)	2 799 404 (18.2)	1 898 104 (16.9)
Not in a patient enrolment model	17 098 (12.4)	412 861 (5.2)	978 529 (6.4)	650 717 (5.8)
Not attributable to a primary care physician	5 001 (3.6)	92 498 (1.2)	150 270 (1.0)	147 885 (1.3)
Rurality				
Large urban	38 141 (27.6)	3 655 502 (45.7)	6 957 659 (45.1)	5 095 877 (45.3)
Urban	32 529 (23.5)	2 734 648 (34.2)	5 443 178 (35.3)	3 804 871 (33.9)
Small urban	54 174 (39.2)	1 168 424 (14.6)	2 347 398 (15.2)	1 626 152 (14.5)
Rural	13 357 (9.7)	432 623 (5.4)	672 771 (4.4)	714 357 (6.4)
Johns Hopkins resource utilization bands				
0 (lowest user)	4779 (3.5)	65 416 (0.8)	229 198 (1.5)	106 843 (1.0)
1	5470 (4.0)	108 597 (1.4)	366 466 (2.4)	166 095 (1.5)
2	20 657 (15.0)	647 359 (8.1)	1 743 956 (11.3)	938 716 (8.4)
3	71 295 (51.6)	4 176 124 (52.3)	8 194 889 (53.1)	5 842 130 (52.0)
4	25 041 (18.1)	1 968 456 (24.6)	3 375 539 (21.9)	2 784 788 (24.8)
5 (highest user)	10 959 (7.9)	1 025 245 (12.8)	1 510 958 (9.8)	1 402 685 (12.5)

Note: COVID-19 = coronavirus disease 2019.

care physicians (*n* = 9572), and the number of visits by Ontarians according to visit location and physician characteristics, are summarized in Table 2.

In the pre-COVID-19 period (Mar. 12 to July 29, 2019), there were 15 421 006 total visits (mean 7.66 visits per 1000 people/d) including 15 174 126 office visits (mean 7.53 visits per 1000 people/d, 98.4% of all visits), 108 679 home visits (mean 0.05 visits per 1000 people/d, 0.7% of all visits), and 138 201 video visits via the Ontario Telemedicine Network (mean 0.07 visits per 1000 people/d, 1.2% of all visits). In the COVID-19 period (Mar. 11 to July 28, 2020), total visits were 28.0% lower than before COVID-19; office visits were 79.1% lower than in the pre-COVID-19 period, and virtual visits constituted 71.1% of all visits (Table 3).

Comparing the COVID-19 period with the corresponding period in 2019, total visits declined by 28.0%, but with relatively smaller decreases among women (26.3%), older adults (23.9% among those age 65–74 yr and 19.1% among those age ≥ 75 yr), long-term OHIP registrants (25.1%), patients who could not be attributed to a primary care physician (10.2%) and patients with high expected health care use (18.2% among those in the second highest group and 8.3% among those with the highest expected use). Rural residents had a 6.4% increase in total visits. The greatest declines were among children (50.5%) and those with low expected health care use (55.7%) (Table 3).

When evaluating the change in total visits by physician characteristics, which decreased by 27.1% overall, there were relatively lower decreases among patients seeing female physicians (25.3%), older physicians (20.8% among those age 65–74 yr and 20.1% among those age ≥ 75 yr), Canadian graduates (24.7%) and those with large panel sizes (21.0% for those with ≥ 2500 patients) (Table 4).

Virtual care constituted 71.1% of all visits in the COVID-19 period, with higher proportions of virtual care visits among women (72.4%), adults aged 65–74 years (73.4%), those in the highest income quintile (74.3%), long-term OHIP registrants (72.8%), patients cared for in Family Health Teams (75.7%), and those with the highest expected health care needs (73.1%). The lowest use of virtual care was among children (57.6% of all visits), those not in a patient enrolment model (63.4%), those who could not be attributed to a primary care physician (62.5%), rural residents (60.6%) and those with the lowest expected health care use (61.2%) (Table 3). Higher proportions of virtual care were provided by female physicians (75.4%) and physicians aged 30–44 years (75.0%) (Table 4).

Weekly counts of visits are depicted in Figure 1, showing a precipitous decline in office visits and a large increase in virtual visits in mid-March 2020. The lowest number of office visits occurred in mid-April; by late July, the numbers had more than tripled, but still remained far below the pre-COVID-19 period. Total visits gradually increased from mid-April onward but remained lower throughout the COVID-19 period than in 2019, reaching 83.4% of the 2019 level by the end of the time period.

Table 2 (part 1 of 2): Number of primary care physicians and visits, Mar. 12 to July 29, 2019 (pre-COVID-19) and Mar. 11 to July 28, 2020 (COVID-19), by visit location and physician characteristics

	No. (%) of physicians*		No. (%) of office visits		No. (%) of home visits	
Variable	Pre-COVID-19 *n* = 9572	COVID-19 *n* = 9572	Pre-COVID-19 *n* = 15 174 126	COVID-19 *n* = 3 206 492	Pre-COVID-19 *n* = 108 679	COVID-19 *n* = 43 568
Physician sex						
Female	4737 (49.5)	4737 (49.5)	6 234 943 (41.1)	1 149 541 (35.8)	37 705 (34.7)	12 441 (28.6)
Male	4835 (50.5)	4835 (50.5)	8 939 183 (58.9)	2 056 951 (64.2)	70 974 (65.3)	31 127 (71.4)
Physician age, yr						
30–44	3187 (33.3)	3187 (33.3)	4 555 425 (30.0)	779 737 (24.3)	31 931 (29.4)	8 784 (20.2)
45–64	4604 (48.1)	4604 (48.1)	8 158 405 (53.8)	1 820 863 (56.8)	53 656 (49.4)	24 726 (56.8)
65–74	1392 (14.5)	1392 (14.5)	1 985 398 (13.1)	514 083 (16.0)	18 718 (17.2)	8 044 (18.5)
≥ 75	333 (3.5)	333 (3.5)	306 146 (2.0)	82 167 (2.6)	2 950 (2.7)	1 911 (4.4)
Graduation country						
Canada	5296 (55.3)	5296 (55.3)	7 103 675 (46.8)	1 535 005 (47.9)	57 673 (53.1)	27 059 (62.1)
Other	2422 (25.3)	2422 (25.3)	5 260 641 (34.7)	1 138 036 (35.5)	30 163 (27.8)	9521 (21.8)
N/A†	1854 (19.4)	1854 (19.4)	2 809 810 (18.5)	533 451 (16.6)	20 84 (19.2)	6988 (16.0)
Physician panel size						
< 100	664 (6.9)	664 (6.9)	362 578 (2.4)	44 474 (1.4)	7602 (7.0)	2171 (5.0)
100–499	1062 (11.1)	1062 (11.1)	1 035 013 (6.8)	179 433 (5.6)	14 513 (13.4)	6613 (15.2)
500–999	2000 (20.9)	2000 (20.9)	2 302 272 (15.2)	433 750 (13.5)	17 668 (16.3)	6154 (14.1)
1000–1499	2666 (27.8)	2666 (27.8)	3 826 043 (25.2)	753 013 (23.5)	28 707 (26.4)	11 133 (25.6)
1500–1999	1749 (18.3)	1749 (18.3)	3 294 063 (21.7)	711 382 (22.2)	20 684 (19.0)	8753 (20.1)
2000–2499	838 (8.8)	838 (8.8)	2 091 176 (13.8)	476 475 (14.9)	13 261 (12.2)	6224 (14.3)
≥ 2500	591 (6.2)	591 (6.2)	2 262 912 (14.9)	607 962 (19.0)	6244 (5.8)	2520 (5.8)

Table 2 (part 2 of 2): Number of primary care physicians and visits, Mar. 12 to July 29, 2019 (pre-COVID-19) and Mar. 11 to July 28, 2020 (COVID-19), by visit location and physician characteristics

Variable	No. (%) of virtual visits		No. (%) of overall visits	
	Pre-COVID-19 *n* = 138 201	COVID-19 *n* = 7 991 197	Pre-COVID-19 *n* = 15 421 006	COVID-19 *n* = 11 241 257
Physician sex				
Female	53 789 (38.9)	3 566 819 (44.6)	6 326 437 (41.0)	4 728 801 (42.1)
Male	84 412 (61.1)	4 424 378 (55.4)	9 094 569 (59.0)	6 512 456 (57.9)
Physician age, yr				
30–44	82 753 (59.9)	2 370 048 (29.7)	4 670 109 (30.3)	3 158 569 (28.1)
45–64	35 893 (26.0)	4 358 367 (54.5)	8 247 954 (53.5)	6 203 956 (55.2)
65–74	3942 (2.8)	1 067 717 (13.4)	2 008 058 (13.0)	1 589 844 (14.1)
≥ 75	58 (0.0)	162 986 (2.0)	309 154 (2.0)	247 064 (2.2)
Graduation country				
Canada	25 751 (18.6)	3 852 638 (48.2)	7 187 099 (46.6)	5 414 702 (48.2)
Other	41 202 (29.8)	2 677 533 (33.5)	5 332 006 (34.6)	3 825 090 (34.0)
N/A†	71 248 (51.6)	1 461 026 (18.3)	2 901 901 (18.8)	2 001 465 (17.8)
Physician panel size				
< 100	2683 (1.9)	117 181 (1.5)	372 863 (2.4)	163 826 (1.5)
100–499	23 737 (17.2)	363 001 (4.5)	1 073 263 (7.0)	549 047 (4.9)
500–999	25 659 (18.6)	1 216 963 (15.2)	2 345 599 (15.2)	1 656 867 (14.7)
1000–1499	19 757 (14.3)	2 137 171 (26.7)	3 874 507 (25.1)	2 901 317 (25.8)
1500–1999	27 328 (19.8)	1 812 409 (22.7)	3 342 075 (21.7)	2 532 544 (22.5)
2000–2499	10 354 (7.5)	1 138 840 (14.2)	2 114 791 (13.7)	1 621 539 (14.4)
≥ 2500	28 683 (20.8)	1 205 632 (15.1)	2 297 839 (14.9)	1 816 114 (16.2)

Note: COVID-19 = coronavirus disease 2019, N/A = not available.
*ICES Physician database is only updated to 2018/19 and was used for both the 2019 and 2020 analyses.
†Data were not available for recent time periods.

Interrupted time series analyses comparing Apr. 1–May 26, 2020, with May 27–July 28, 2020, showed that average daily visits increased significantly by 1509 visits per week (95% CI 957–2061) in the earlier time period, with a significantly lower increase of 517 visits per week (95% CI 55–979) in the later time period. There was an upward trend in average daily office visits that was significantly greater in the later time period, with an increase of 993 visits per week (95% CI 778–1208) in the earlier time period and 1410 per week (95% CI 1230–1590) in the later time period. Virtual visits increased significantly in the earlier COVID-19 period (512 visits per week, 95% CI 132–892), but declined significantly in the later COVID-19 period (897 visits per week, 95% CI 579–1214) (see Appendix 2 for details, available at www.cmaj.ca/lookup/doi/10.1503/cmaj.202303/tab-related-content).

Interpretation

We describe sudden, striking shifts in primary care patterns in Ontario in the early months of the COVID-19 pandemic, when physical distancing directives necessitated shifts in the way health care was delivered. We found an almost 80% decrease in office visits and a 56-fold increase in virtual visits, changing most dramatically in mid-March 2020. Although it will take time to fully understand the impact of these changes and their effects on different groups, our early findings provide some reassurance that the groups with the highest care needs, including those older than 65 years and those with higher levels of morbidity, maintained relatively higher levels of care overall. Virtual care increased markedly for all groups, with relatively small differences across patient and physician characteristics.

Trends over time showed some recovery of office visits after the initial precipitous decline, but not back to the previous year's baseline by the end of July 2020. The COVID-19 period, from mid-March to the end of July 2020, was not a homogeneous period, with gradual and regional lifting of restrictions from early May to mid-July.[12] Trends showed increasing office visits over this time period, with greater increases in June and July, along with decreases in virtual care. Although valuable services were undoubtedly lost, it is likely that unnecessary visits and low-value care were also reduced. The lower levels of virtual care seen among children and in rural areas may warrant further attention.

Other jurisdictions have also reported decreases in the use of primary and ambulatory care, and rapid increases in virtual care during the early phases of the COVID-19 pandemic.[13–16] A large decrease in physician services was documented in 3 Canadian provinces in March and April, with subsequent increases in May and June 2020,[17] and a partial rebound in services after a decline has

Table 3 (part 1 of 2): Mean number of primary care visits, Mar. 12 to July 29, 2019 (pre–COVID-19) and Mar. 11 to July 28, 2020 (COVID-19), by visit location and patient characteristics

	No. office visits per 1000 people/d*			No. home visits per 1000 people/d†				No. virtual visits per 1000 people/d*		
Variable	Pre-COVID-19	COVID-19	Change, %	Pre-COVID-19	COVID-19	Change, %	*p* value	Pre-COVID-19	COVID-19	Change, %
Total	7.53	1.57	−79.1	0.05	0.02	−60.4		0.07	3.92	5608.4
Sex										
Female	8.59	1.75	−79.6	0.07	0.03	−61.3		0.08	4.66	5923.1
Male	6.44	1.39	−78.4	0.04	0.02	−59.0		0.06	3.15	5189.9
Age, yr										
≤ 18	5.05	1.07	−78.8	0.02	0.00	−92.7		0.05	1.46	3054.0
19–29	6.22	1.33	−78.7	0.01	0.00	−81.9	0.002	0.09	3.10	3195.2
30–44	6.97	1.45	−79.2	0.01	0.00	−84.0		0.08	3.74	4613.9
45–64	8.30	1.71	−79.4	0.02	0.01	−65.4		0.07	4.70	6570.6
65–74	10.37	2.09	−79.8	0.07	0.03	−51.5		0.06	5.87	9026.1
≥ 75	11.51	2.47	−78.5	0.42	0.19	−54.5		0.05	7.04	13 554.0
Neighbourhood income quintile										
Q1 (lowest)	7.66	1.73	−77.5	0.06	0.03	−56.4		0.08	3.90	4585.4
Q2	7.78	1.61	−79.3	0.06	0.02	−60.8		0.07	4.01	5466.1
Q3	7.78	1.54	−80.3	0.05	0.02	−60.2		0.07	3.98	6010.6
Q4	7.48	1.40	−81.2	0.05	0.02	−62.2		0.07	3.81	5580.5
Q5 (highest)	6.95	1.24	−82.2	0.05	0.02	−63.8		0.06	3.64	6427.2
Recent registrant < 10 yr										
No	7.64	1.56	−79.6	0.06	0.03	−57.0		0.07	4.23	5881.6
Yes	8.15	1.66	−79.6	0.02	0.00	−71.7	0.004	0.06	3.82	6031.4
Model of primary care										
Capitation	7.30	1.31	−82.1	0.05	0.02	−55.2		0.05	3.64	6645.1
Enhanced fee-for-service	10.85	2.44	−77.5	0.07	0.03	−61.7		0.08	6.01	7386.7
Family Health Team	5.48	0.90	−83.7	0.04	0.02	−57.4		0.06	2.85	4334.6
Not in at patient enrolment model	6.56	1.61	−75.4	0.08	0.02	−68.2		0.12	2.85	2310.1
Not attributable to a primary care physician	0.90	0.31	−65.2	0.01	0.00	−67.8	0.018	0.03	0.53	1586.9
Rurality										
Large urban	8.07	1.64	−79.7	0.06	0.03	−57.0		0.04	4.22	9320.2
Urban	8.80	1.71	−80.6	0.05	0.02	−70.8		0.05	4.41	8183.7
Small urban	5.87	1.15	−80.4	0.05	0.02	−51.9		0.14	2.99	2042.6
Rural	3.96	1.70	−57.2	0.05	0.02	−58.0	< 0.001	0.08	2.64	3146.3
Johns Hopkins resource utilization bands										
0 (lowest user)	1.05	0.18	−82.5	0.00	0.00	−64.2	0.434	0.02	0.29	1201.4
1	2.88	0.46	−84.1	0.00	0.00	−82.3	0.118	0.04	0.87	1878.6
2	4.26	0.72	−83.2	0.01	0.00	−81.5	< 0.001	0.05	1.59	3014.8
3	8.73	1.77	−79.7	0.03	0.01	−68.6		0.08	4.46	5701.6
4	13.17	3.17	−76.0	0.10	0.04	−60.2		0.10	7.73	7697.4
5 (highest user)	15.57	3.75	−75.9	0.53	0.25	−53.8		0.12	10.87	9138.2

Table 3 (part 2 of 2): Mean number of primary care visits, Mar. 12 to July 29, 2019 (pre–COVID-19) and Mar. 11 to July 28, 2020 (COVID-19), by visit location and patient characteristics

	No. overall visits per 1000 people/d†				% virtual
**Variable**	**Pre-COVID-19**	**COVID-19**	**Change, %**	***p* value**	**COVID-19**
Total	7.66	5.51	−28.0		71.1
Sex					
Female	8.73	6.43	−26.3		72.4
Male	6.54	4.56	−30.3		69.1
Age, yr					
≤ 18	5.12	2.53	−50.5		57.6
19–29	6.32	4.43	−30.0		70.0
30–44	7.06	5.19	−26.5		72.0
45–64	8.40	6.42	−23.5		73.2
65–74	10.51	8.00	−23.9		73.4
≥ 75	11.99	9.70	−19.1		72.5
Neighbourhood income quintile					
Q1 (lowest)	7.80	5.65	−27.6		69.0
Q2	7.91	5.65	−28.6		71.1
Q3	7.89	5.53	−29.9		71.9
Q4	7.59	5.23	−31.1		72.8
Q5 (highest)	7.05	4.90	−30.6		74.3
Recent registrant < 10 yr					
No	7.77	5.82	−25.1		72.8
Yes	8.23	5.48	−33.4		69.6
Model of primary care					
Capitation	7.40	4.96	−32.9		73.3
Enhanced fee for-service	11.00	8.47	−23.0		70.9
Family Health Team	5.59	3.77	−32.6		75.7
Not in at patient enrolment model	6.76	4.49	−33.6		63.4
Not attributable to a primary care physician	0.94	0.85	−10.2	0.013	62.5
Rurality					
Large urban	8.17	5.88	−28.0		71.7
Urban	8.91	6.14	−31.1		71.9
Small urban	6.05	4.16	−31.2		71.9
Rural	4.10	4.36	6.4	0.002	60.6
Johns Hopkins resource utilization bands					
0 (lowest user)	1.08	0.48	−55.7		61.2
1	2.93	1.32	−54.8		65.4
2	4.32	2.31	−46.5		69.0
3	8.84	6.24	−29.4		71.5
4	13.37	10.94	−18.2		70.7
5 (highest user)	16.22	14.87	−8.3		73.1

Note: COVID-19 = coronavirus disease 2019.
*All p values for visit types are < 0.0001.
†p values are < 0.0001 unless indicated otherwise. The p value compares the number of daily visits per 1000 people in the pre-COVID-19 versus COVID-19 periods.

Table 4: Mean number of primary care visits, Mar. 12 to July 29, 2019 (pre–COVID-19) and Mar. 11 to July 28, 2020 (COVID-19), by visit location and physician characteristics

	No. office visits per physician/d*			No. home visits per physician/d*†			No. virtual visits per physician/d*			No. overall visits per physician/d*			% virtual
Variable	Pre-COVID-19	COVID-19	Change, %	Pre-COVID-19	COVID-19	Change, %	Pre-COVID-19	COVID-19	Change, %	Pre-COVID-19	COVID-19	Change, %	COVID-19
Total	11.32	2.39	−78.9	0.08	0.03	−59.9	0.10	5.96	5682.3	11.51	8.39	−27.1	71.1
Physician sex													
Female	9.40	1.73	−81.6	0.06	0.02	−67.0	0.08	5.38	6531.1	9.54	7.13	−25.3	75.4
Male	13.21	3.04	−77.0	0.10	0.05	−56.1	0.12	6.54	5141.4	13.44	9.62	−28.4	67.9
Physician age, yr													
30–44	10.21	1.75	−82.9	0.07	0.02	−72.5	0.19	5.31	2764.0	10.47	7.08	−32.4	75.0
45–64	12.66	2.82	−77.7	0.08	0.04	−53.9	0.06	6.76	12 042.7	12.80	9.63	−24.8	70.3
65–74	10.19	2.64	−74.1	0.10	0.04	−57.0	0.02	5.48	26 985.7	10.30	8.16	−20.8	67.2
≥ 75	6.57	1.76	−73.2	0.06	0.04	−35.2	0.00	3.50	280 910.3	6.63	5.30	−20.1	66.0
Graduation country													
Canada	9.58	2.07	−78.4	0.08	0.04	−53.1	0.03	5.20	14 861.1	9.69	7.30	−24.7	71.2
Other	15.51	3.36	−78.4	0.09	0.03	−68.4	0.12	7.90	6398.6	15.72	11.28	−28.3	70.0
N/A‡	10.83	2.06	−81.0	0.08	0.03	−66.5	0.27	5.63	1950.6	11.18	7.71	−31.0	73.0
Physician panel size													
< 100	3.90	0.48	−87.7	0.08	0.02	−71.4	0.03	1.26	4267.5	4.01	1.76	−56.1	71.5
100–499	6.96	1.21	−82.7	0.10	0.04	−54.4	0.16	2.44	1429.3	7.22	3.69	−48.8	66.1
500–999	8.22	1.55	−81.2	0.06	0.02	−65.2	0.09	4.35	4642.8	8.38	5.92	−29.4	73.4
1000–1499	10.25	2.02	−80.3	0.08	0.03	−61.2	0.05	5.73	10 717.3	10.38	7.77	−25.1	73.7
1500–1999	13.45	2.91	−78.4	0.08	0.04	−57.7	0.11	7.40	6532.1	13.65	10.34	−24.2	71.6
2000–2499	17.82	4.06	−77.2	0.11	0.05	−53.1	0.09	9.71	10 899.0	18.03	13.82	−23.3	70.2
≥ 2500	27.35	7.35	−73.1	0.08	0.03	−59.6	0.35	14.57	4103.3	27.77	21.95	−21.0	66.4

Note: COVID-19 = coronavirus disease 2019, N/A = not available.
*All *p* values for visit types are < 0.0001 unless indicated otherwise. The *p* value compares the number of daily visits per physician in the pre-COVID-19 versus COVID-19 periods.
†The *p* value for variable "Physician ≥ age 75 yr" under the category "No. home visits per physician/d" is $p = 0.2079$.
‡Data were not available for recent time periods.

also been seen in the US.[18] One study noted that blood pressure and cholesterol assessments, and new medication visits, declined substantially.[19] Most studies described overall trends and did not assess equity in changes in access, although pre-COVID-19 studies have documented inequitable access to virtual care.[20]

Telemedicine and virtual care have been established in Canadian health care for decades, especially for Northern and remote regions, but until 2020, virtual visits comprised a very small proportion of all care. Virtual care has the advantages of reducing the impact of health care delivery on the environment and eliminating the potential for exposure to pathogens such as severe acute respiratory syndome coronavirus 2. Its disadvantages include the inability to perform most physical examinations or procedures, difficulty establishing new therapeutic relationships, dealing with some complex mental health issues, missing body language and nonverbal cues and lacking the full degree of comfort and support that can be provided in person. Educational attainment, digital literacy, age, rurality, language and culture all contribute to the "digital divide."[20,21] Concerns have been raised about virtual visits, including privacy, continuity of care and equity of implementation.[21,22] An Ontario pilot study of virtual care showed that, when provided with a choice of audio, video or text messaging on an integrated platform, more than 90% of visits occurred using asynchronous, secure text messaging followed by audio, yet there are no billing codes for text messaging.[28]

Despite extensive use of virtual care in the COVID-19 pandemic,[23–27] the appropriate role of virtual care remains to be determined. It is not possible to separate the role of physician funding for virtual care from the impact of the pandemic itself in the major uptake of virtual care. However, the pandemic lockdown was undoubtedly an important driver of the large decline in office visits. The longer-term prospects for funding of virtual care, including which modalities are funded, likely rest on its impact on access to care, quality of care and costs, all of which hold promise but require further investigation and policy development. How care is provided also has large workforce implications that require further exploration. Canadians appear to be highly satisfied with virtual care and up to one-third would like virtual care to be the first point of contact after the pandemic.[29] There is support for virtual care to be covered by employer health plans, posing challenges to continuity of care and equity, if virtual care is not publicly funded in the future.[30]

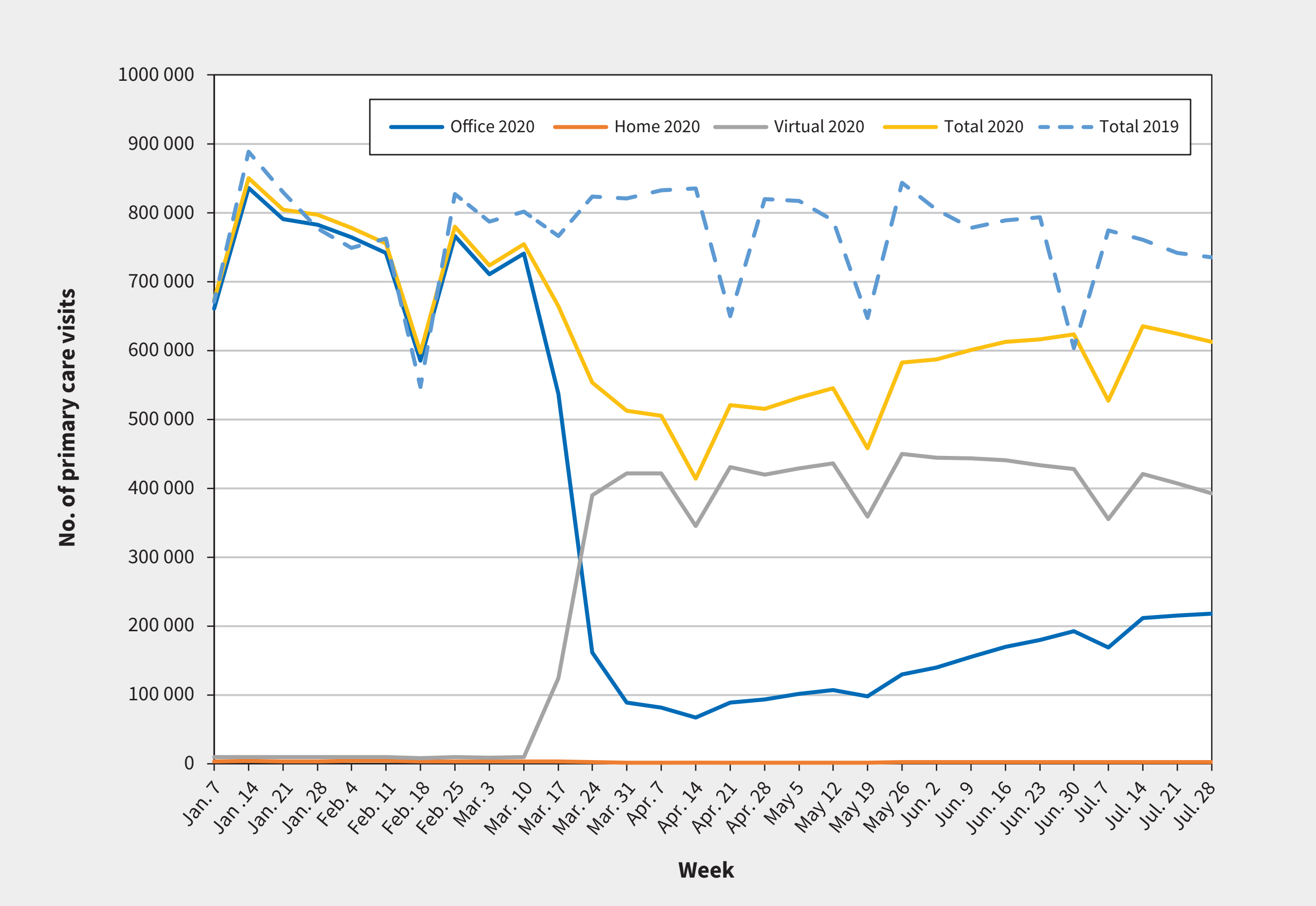

Figure 1: Weekly primary care total visits and visits by type (office, home or virtual), January to July 2020, and total visits, January to July 2019, Ontario, Canada.

Limitations

Strengths of this study include its population-wide coverage and use of recent data, but it also has several limitations. We did not assess reasons for visits and therefore could not assess the need or value of forgone visits. It was not possible to separate telephone visits from video visits using temporary billing codes, and we could not assess the use of email or secure messaging because no billing codes were available for those services. The extent of care provided by phone before the pandemic is not well understood, so the increase in virtual care may have been overestimated. The Johns Hopkins Adjusted Clinical Groups system relies on health care use, so the disruption in care during the COVID-19 pandemic could have affected these measures. Billings for July 2020 were those submitted in August 2020, so some incompleteness is expected, especially for the final week of July. COVID-19 has disproportionately affected racialized communities, and the lack of routine data collection about ethnicity hinders our further understanding of these disparities, including forgone care and the use of virtual care.[31] The available data do not include primary care provided by nonphysicians. A greater understanding of care provision by primary care providers is needed, including the reasons for changes in care and an assessment of physicians who may have completely stopped in-person visits or ceased practising entirely. How and why both physicians and patients made choices about modality of care requires further attention, as do reasons for those choices, such as a lack of adequate personal protective equipment. Other Canadian provinces and territories implemented different billing codes to support virtual care, so our findings are not generalizable to other Canadian jurisdictions.

Conclusion

We report preliminary data on the extent to which office and virtual primary care changed during the initial months of COVID-19, and how this varied by type of patients and physicians. We found sudden and dramatic decreases in office visits and large increases in virtual care, with an overall substantial decrease in care provided. These changes affected patient and physician subgroups differently. The determinants and consequences of these major shifts in care, and for which patients and providers, require further study.

References

1. Stewart M, Ryan B. Ecology of health care in Canada. *Can Fam Physician* 2015;61:449-53.
2. Starfield B, Shi L, Macinko J. Contribution of primary care to health systems and health. *Milbank Q* 2005;83:457-502.
3. WHO characterizes COVID-19 as a pandemic [news release]. 2020 Mar. 11. Geneva: The World Health Organization; 2020. Available: www.who.int/emergencies/diseases/novel-coronavirus-2019/events-as-they-happen (accessed 2020 Sept. 22).
4. COVID-19 intervention timeline in Canada. Ottawa: Canadian Institute for Health Information; 2020. Available: www.cihi.ca/en/covid-19-intervention-timeline-in-canada (accessed 2020 Sept. 22).
5. Quick COVID-19 primary care survey of clinicians: summary of the eleventh (August 21–24, 2020) pan-Canadian survey of frontline primary care clinicians' experience with COVID-19. Ottawa: Canadian Institutes of Health Research

(Primary and Integrated Health Care Innovation Network); 2020. Available: http://spor-pihci.com/wp-content/uploads/2020/09/PIHCIN-Covid-19-survey-report-11.pdf (accessed 2020 Sept. 22).
6. COVID-19 Collection: Primary Care Covid-19 Survey: Larry Green Center and Primary Care Collaborative. *Annal Fam Med* 2020. Available: https://www.annfammed.org/content/covid-19-collection-primary-care-covid-19-survey-larry-green-center-and-primary-care (accessed 2020 Sept. 22).
7. Ontario Demographic Quarterly: highlights of first quarter 2020. Toronto: Government of Ontario; 2020. Available: www.ontario.ca/page/ontario-demographic-quarterly-highlights-first-quarter-2020 (accessed 2020 Sept. 22).
8. Schultz SE, Glazier RH. Identification of physicians providing comprehensive primary care in Ontario: a retrospective analysis using linked administrative data. *CMAJ Open* 2017;5:E856-63.
9. Kralj B. Measuring rurality — RIO2008 BASIC: methodology and results Toronto: Ontario Medical Association; 2009. Available: https://content.oma.org//wp-content/uploads/2008rio-fulltechnicalpaper.pdf (accessed 2020 Sept. 22).
10. Hutchison B, Glazier R. Ontario's primary care reforms have transformed the local care landscape, but a plan is needed for ongoing improvement. *Health Aff (Millwood)* 2013;32:695-703.
11. The Johns Hopkins ACG system. Baltimore: Johns Hopkins University. Available: www.hopkinsacg.org/ (accessed 2020 Sept. 22).
12. Nielsen K. A timeline of the novel coronavirus in Ontario. *Global News* 2020 Apr. 24. Available: https://globalnews.ca/news/6859636/ontario-coronavirus-timeline/ (accessed 2020 Nov. 23).
13. Basu S, Phillips RS, Phillips R, et al. Primary care practice finances in the United States amid the COVID-19 pandemic. *Health Aff (Millwood)* 2020;39:1605-14.
14. Healthcare professionals and the impact of COVID-19: a comparative study of revenue and utilization [FAIR Health briefs]. New York: Fair Health; 2020 June 10. Available: www.fairhealth.org/publications/briefs (accessed 2020 Sept. 22).
15. Heintzman J, O'Malley J, Marino M, et al. SARS-CoV-2 testing and changes in primary care services in a multistate network of community health centers during the COVID-19 pandemic. *JAMA* 2020 Aug 31;e2015891. doi: 10.1001/jama.2020.15891. [Epub ahead of print].
16. How might COVID-19 have affected people's ability to see their GP? London (UK): The Health Foundation; 2020. Available: www.health.org.uk/news-and-comment/charts-and-infographics/how-might-covid-19-have-affected-peoples-ability-to-see-GP (accessed 2020 Sept. 22).
17. How COVID-19 affected physician services. Ottawa: Canadian Institute for Health Information; 2020. Available: www.cihi.ca/en/covid-19-resources/impact-of-covid-19-on-canadas-health-care-systems/how-covid-19-affected-physician-services (accessed 2020 Nov. 23).
18. Mehrotra A, Chernew M, Linetsky D, et al. The impact of the COVID-19 pandemic on outpatient visits: a rebound emerges. New York: Commonwealth Fund; 2020. Available: www.commonwealthfund.org/publications/2020/apr/impact-covid-19-outpatient-visits (accessed 2020 Sept. 22).
19. Alexander GC, Tajanlangit M, Heyward J, et al. Use and content of primary care office-based vs telemedicine care visits during the COVID-19 pandemic in the US. *JAMA Netw Open* 2020;3:e2021476.
20. *Household access to the Internet at home, by household income quartile and geography*. Ottawa: Statistics Canada; 2020. Available: www150.statcan.gc.ca/t1/tbl1/en/tv.action?pid=2210000701 (accessed 2020 Sept. 22).
21. Hardcastle L, Ogbogu U. Virtual care: enhancing access or harming care? *Healthc Manage Forum* 2020 Nov;33:288-92.
22. Ortega G, Rodriguez JA, Maurer LR, et al. Telemedicine, COVID-19, and disparities: Policy implications. *Health Policy Technol* 2020;9:368-71.
23. Srinivasan M, Asch S, Vilendrer S, et al. Qualitative assessment of rapid system transformation to primary care video visits at an academic medical. *Ann Intern Med* 2020;173:527-35.
24. Lau J, Knudsen J, Jackson H, et al. Staying connected in the COVID-19 pandemic: telehealth at the largest safety-net system in the United States. *Health Aff (Millwood)* 2020;39:1437-42.
25. Olayiwola JN, Magaña C, Harmon A, et al. Telehealth as a bright spot of the COVID-19 pandemic: Recommendations from the virtual frontlines ("Frontweb"). *JMIR Public Health Surveill* 2020;6:e19045.
26. Dewar S, Lee PG, Suh TT, et al. Uptake of virtual visits in a geriatric primary care clinic during the COVID-19 pandemic. *J Am Geriatr Soc* 2020;68:1392-4.
27. Nouri S, Khoong EC, Lyles CR, et al. Addressing equity in telemedicine for chronic disease management during the Covid-19 pandemic. *NEJM Catal* 2020 May 4.
28. Enhanced access to primary care: project evaluation final report. Toronto: Women's College Hospital Institute for Health Systems Solutions and Virtual Care (WIHV); 2019. Available: https://otn.ca/wp-content/uploads/2019/08/eapc-evaluation-report.pdf (accessed 2020 Sept. 22).
29. What Canadians think about virtual health care? Ottawa: Abacus Data/Canadian Medical Association; 2020. Available: https://abacusdata.ca/wp-content/uploads/2020/06/CMA-Abacus-May-2020-Jun-4-ENv2.pdf (accessed 2020 Dec. 13).
30. Canadian attitudes on healthcare and telemedicine. Environics Research. Montréal and Toronto: Dialogue; 2020. Available: www.dialogue.co/canadian-attitudes-on-healthcare-and-telemedicine (accessed 2020 Dec. 13).
31. Sivashanker K, Duong T, Ford S, et al. A data-driven approach to addressing racial disparities in health care outcomes. *Harv Bus Rev* 2020 July 21. Available: hbrorg/2020/07/a-data-driven-approach-to-addressing-racial-disparities-in-health-care-outcomes (accessed 2020 Dec. 13).

Competing interests: None declared.

This article has been peer reviewed.

Affiliations: ICES Central (Glazier, Wu, Kopp, Kiran); Primary Care and Health Systems (Glazier, Kiran, Kopp); St. Michael's Hospital Centre for Urban Health Solutions (Glazier, Kiran), Toronto, Ont.; Department of Family Medicine, and Health Services and Policy Research Institute (Green), Queen's University, Kingston, Ont.; Health Services and Policy Research Institute, Queen's University, and ICES Queen's (Frymire), Kingston, Ont.; Department of Family and Community Medicine (Glazier, Kiran), St. Michael's Hospital, University of Toronto, Toronto, Ont.

Contributors: Richard Glazier and Tara Kiran conceived of the study and drafted the manuscript. All of the authors designed the study. Fangyun Wu and Alex Kopp analyzed the data. All of the authors interpreted the data, revised the manuscript critically for important intellectual content, approved the final version to be published and agreed to be accountable for all aspects of the work.

Content licence: This is an Open Access article distributed in accordance with the terms of the Creative Commons Attribution (CC BY-NC-ND 4.0) licence, which permits use, distribution and reproduction in any medium, provided that the original publication is properly cited, the use is noncommercial (i.e., research or educational use), and no modifications or adaptations are made. See: https://creativecommons.org/licenses/by-nc-nd/4.0/

Funding: This work was supported by INSPIRE-PHC and by ICES, both of which are funded by grants from the Ontario Ministry of Health and Long-Term Care. This work was also supported by the Canadian Institutes of Health Research, funding reference number SOP 162662. Richard Glazier and Tara Kiran are supported as Clinician Scientists by the Department of Family and Community Medicine at the University of Toronto and at St. Michael's Hospital. Tara Kiran is the Fidani Chair in Improvement and Innovation at the University of Toronto. Michael Green is supported by the Brian Hennen Chair in Family Medicine at Queen's University.

Data sharing: The data set from this study is held securely in coded form at ICES. Although data sharing agreements prohibit ICES from making the data set publicly available, access may be granted to those who meet prespecified criteria for confidential access, available at https://www.ices.on.ca/DAS. The full data set creation plan and underlying analytic code are available from the authors on request, with the understanding that the computer programs may rely on coding templates or macros that are unique to ICES and are therefore inaccessible or may require modification.

Acknowledgements: The authors thank Lidija Latifovic from the University of Toronto, Dalla Lana School of Public Health, Epidemiology, for her assistance with review of the literature.

Disclaimer: This study was supported by ICES, which is funded by an annual grant from the Ontario Ministry of Health and Long-Term Care (MOHLTC). The opinions, results and conclusions reported in this paper are those of the authors and are independent from the funding sources. No endorsement by ICES or the Ontario MOHLTC is intended or should be inferred.

Accepted: Dec. 23, 2020

Correspondence to: Richard Glazier, rick.glazier@ices.on.ca
