# Supplementary material for: Changes in the top 25 reasons for primary care visits during the COVID-19 pandemic in a high-COVID region of Canada
Source: PLoS One. 2021 Aug 12;16(8):e0255992. doi: 10.1371/journal.pone.0255992 (PMC8360367; doi:10.1371/journal.pone.0255992)
Supplement: Attachment — Submitted filename: Primary Care Telemedicine visits US (JAMA Oct 2020).pdf [file pone.0255992.s006.pdf]

JAMA Network Open

Original Investigation | Health Policy

Use and Content of Primary Care Office-Based vs Telemedicine Care Visits During the COVID-19 Pandemic in the US

G. Caleb Alexander, MD, MS; Matthew Tajanlangit; James Heyward, MPH; Omar Mansour, MHS; Dima M. Qato, PharmD, MPH, PhD; Randall S. Stafford, MD, PhD

Abstract

IMPORTANCE Little is known about the association between the coronavirus disease 2019 (COVID-19) pandemic and the level and content of primary care delivery in the US.

OBJECTIVE To quantify national changes in the volume, type, and content of primary care delivered during the COVID-19 pandemic, especially with regard to office-based vs telemedicine encounters.

DESIGN, SETTING, AND PARTICIPANTS Analysis of serial cross-sectional data from the IQVIA National Disease and Therapeutic Index, a 2-stage, stratified nationally representative audit of outpatient care in the US from the first calendar quarter (Q1) of 2018 to the second calendar quarter (Q2) of 2020.

MAIN OUTCOMES AND MEASURES Visit type (office-based or telemedicine), overall and stratified by patient population and geographic region; assessment of blood pressure or cholesterol measurement; and initiation or continuation of prescription medications.

RESULTS In the 8 calendar quarters between January 1, 2018, and December 31, 2019, between 122.4 million (95% CI, 117.3-127.5 million) and 130.3 million (95% CI, 124.7-135.9 million) quarterly primary care visits occurred in the US (mean, 125.8 million; 95% CI, 121.7-129.9 million), most of which were office-based (92.9%). In 2020, the total number of encounters decreased to 117.9 million (95% CI, 112.6-123.2 million) in Q1 and 99.3 million (95% CI, 94.9-103.8 million) in Q2, a decrease of 21.4% (27.0 million visits) from the average of Q2 levels during 2018 and 2019. Office-based visits decreased 50.2% (59.1 million visits) in Q2 of 2020 compared with Q2 2018-2019, while telemedicine visits increased from 1.1% of total Q2 2018-2019 visits (1.4 million quarterly visits) to 4.1% in Q1 of 2020 (4.8 million visits) and 35.3% in Q2 of 2020 (35.0 million visits). Decreases occurred in blood pressure level assessment (50.1% decrease, 44.4 million visits) and cholesterol level assessment (36.9% decrease, 10.2 million visits) in Q2 of 2020 compared with Q2 2018-2019 levels, and assessment was less common during telemedicine than during office-based visits (9.6% vs 69.7% for blood pressure; $P < .001$; 13.5% vs 21.6% for cholesterol; $P < .001$). New medication visits in Q2 of 2020 decreased by 26.0% (14.1 million visits) from Q2 2018-2019 levels. Telemedicine adoption occurred at similar rates among White individuals and Black individuals (19.3% vs 20.5% of patient visits, respectively, in Q1/Q2 of 2020), varied by region (low of 15.1% of visits [East North Central region], high of 26.8% of visits [Pacific region]), and was not correlated with regional COVID-19 burden.

CONCLUSIONS AND RELEVANCE The COVID-19 pandemic has been associated with changes in the structure of primary care delivery, with the content of telemedicine visits differing from that of office-based encounters.

JAMA Network Open. 2020;3(10):e2021476. doi:10.1001/jamanetworkopen.2020.21476

Key Points

Question Is there a quantifiable association between the coronavirus disease 2019 (COVID-19) pandemic and the volume, type, and content of primary care encounters in the US?

Findings In this cross-sectional analysis of the US National Disease and Therapeutic Index audit of more than 125.8 million primary care visits in the 10 calendar quarters between quarter 1 of 2018 and quarter 2 of 2020, primary care visits decreased by 21.4% during the second quarter of 2020 compared with the average quarterly visit volume of the second quarters of 2018 and 2019. Evaluations of blood pressure and cholesterol levels decreased owing to fewer total visits and less frequent assessment during telemedicine encounters.

Meaning The COVID-19 pandemic was associated with changes in the structure of primary care delivery during the second quarter of 2020, with the content of telemedicine visits differing from that of office-based encounters.

+ Invited Commentary

+ Supplemental content

Author affiliations and article information are listed at the end of this article.

Open Access. This is an open access article distributed under the terms of the CC-BY License.

Downloaded From: https://jamanetwork.com/ by a University of Manitoba User on 06/09/2021

Introduction

Since February 2020, the coronavirus disease 2019 (COVID-19) pandemic has been associated with more than 4.4 million cases and 150 000 deaths in the US, as well as widespread social and economic changes.[1] While the ultimate health care system impacts of the pandemic remain uncertain, many early health care consequences associated with the pandemic have been noted, ranging from postponement of elective care to permanent clinic and hospital closures.[2,3]

Early reports suggested that substantial increases could be expected in the delivery of telemedicine, or remote clinical services, during the first few months of the pandemic in the US,[4,5] owing to concern regarding the potential for workplace transmission of COVID-19, the implementation of social distancing policies, and the redeployment of health care personnel. A more recent update indicated that the delivery of telemedicine increased during mid-April and has since subsided modestly, although levels remain substantially higher than before the pandemic.[6] These changes, which have been accompanied by changes in federal[7,8] and state[9,10] guidance and reimbursement, have occurred in the context of structural and social factors[11,12] hindering widespread telemedicine adoption.

Investigations of telemedicine during the pandemic, while yielding insights, have generally been based on small or nonrepresentative samples and limited to analyses of the frequency of such encounters rather than descriptions of their content.[4-6,13] We quantified national changes in the volume and type of primary care associated with the COVID-19 pandemic. In addition to characterizing blood pressure and cholesterol measurement and initiation or continuation of prescription medicines for hypertension and dyslipidemia, we explored variance in telemedicine use across different patient populations and geographic regions of the US.

Methods

Data

We used the IQVIA National Disease and Therapeutic Index to conduct a cross-sectional analysis, focusing on the period from the first quarter (Q1) of 2018 through the second quarter (Q2) of 2020. The National Disease and Therapeutic Index is a nationally representative audit of outpatient practice in the US.[14,15] Other studies[16-18] have compared the National Disease and Therapeutic Index with the National Ambulatory Medical Care Survey, a nationally representative audit conducted by the National Center for Health Statistics, and found that the surveys yielded substantively comparable estimates of outpatient care. Using a 2-stage, stratified sampling design, the National Disease and Therapeutic Index audit is based on a sample derived from the American Medical Association and the American Osteopathic Association.[19] Data are collected from approximately 4000 physicians during each calendar quarter, during which participants complete a form for 2 consecutive days documenting each patient encounter, including demographic information, diagnoses, and treatment provided. Diagnostic information is reported using a system similar to the *International Classification of Diseases, Ninth Revision*. The original development of the National Disease and Therapeutic Index was limited to the contiguous US because of statistical sampling and design considerations; as a result, physician reporting from Alaska and Hawaii are excluded. Each encounter is also classified based on site of care, including office-based, hospital-based, telemedicine, or other (such as home, nursing facility, or other institutional setting). Clinician-reported demographic information about individuals, such as patient age, sex, and race, were also assessed. Data were then weighted to provide nationally representative estimates as well as estimates that are representative across 8 geographic regions (Pacific, East South Central, West South Central, West North Central, Mountain, South Atlantic, East North Central, and New England and Mid-Atlantic) (eTable 1 in the Supplement). This study was deemed exempt from institutional review board review by the Johns Hopkins Bloomberg School of Public Health per regulations found at 45 CFR 46. This study followed the

Downloaded From: https://jamanetwork.com/ by a University of Manitoba User on 06/09/2021

Strengthening the Reporting of Observational Studies in Epidemiology (STROBE) reporting guideline for cross-sectional studies.[20]

We restricted our analysis to primary care visits, defined as those accounted for by the fields of internal medicine, pediatrics, geriatrics, general practice, and family practice. For cases in which we specifically assessed pharmaceutical prescribing (eg, initiations of pharmacologic therapy for specific diseases), we examined treatment visit, defined as a patient encounter for a specific diagnosis in which a pharmacologic treatment was initiated or continued. Except where depicted otherwise, we excluded the approximately 3% to 4% of hospital-based visits and 1% to 2% of visits taking place in other settings (home, nursing home, and unspecified sites of care) each calendar quarter.

Our analysis of telemedicine use across geographic regions included an examination of how such use varied by COVID-19 burden, expressed as the rate of COVID-19 fatalities per 100 000 individuals. To estimate this rate, we summed COVID-19 deaths within each national region as of July 28, 2020,[21] and divided this number by the total population within that region.[22]

Statistical Analysis

We used descriptive statistics to perform our analysis. Our main outcomes were visit type, assessment of blood pressure or cholesterol measurement, and initiation or continuation of prescription medicines. First, we extracted the total number of visits between January 1, 2018, and June 30, 2020, and plotted these numbers over time to examine general trends and to assess for inflection points and outliers. Next, we aggregated these visits by calendar quarter. We then characterized the distribution of visits, stratified by encounter type, across visit characteristics of interest, such as patient age, sex, race, and type of insurance. We limited our analysis of telemedicine use by race to Black race and White race given racial disparities in health care. We calculated a Pearson correlation coefficient to examine the association between telemedicine use and COVID-19 burden across 8 geographic regions. We used standardized errors to estimate 95% CIs. Analyses used 2-tailed, unpaired testing. A threshold of $P < .05$ was used to establish statistical significance. Statistical analyses were conducted using Stata software, version 15 (StataCorp LLC).

Results

Trends in Primary Care Visits by Encounter Type

In the 8 calendar quarters between January 1, 2018, and December 31, 2019, between 122.4 million (95% CI, 117.3-127.5 million) and 130.3 million (95% CI, 124.7-135.9 million) quarterly primary care visits occurred in the US (mean, 125.8 million; 95% CI, 121.7-129.9 million) (**Table 1**) (eFigure in the Supplement). In 2020, the total number of encounters decreased to 117.9 million (95% CI, 112.6-123.2 million) in Q1 and 99.3 million (95% CI, 94.9-103.8 million) in Q2, a decrease of 21.4% (27.0 million visits) from the average number of Q2 encounters in 2018 and 2019.

Most primary care encounters in 2018-2019 were office-based (92.9%). Office-based visits decreased from a mean of 116.9 million (95% CI, 111.6-122.1 million) for the average quarterly visit volume in 2018-2019 to 105.9 million (95% CI, 101.2-110.7 million) in Q1 of 2020, then 58.7 million (95% CI, 55.3-62.1 million) in Q2 of 2020, a decrease of 50.2% (59.1 million visits) compared with Q2 2018-2019 levels. By contrast, telemedicine visits increased from 1.1% of visits in 2018-2019 to 4.1% in Q1 of 2020 and 35.3% of visits in Q2 of 2020.

Primary Care Visits by Race and Other Patient Characteristics

Table 2 presents the use of office-based and telemedicine visits stratified by patient race. For example, during the first 2 quarters of 2018, there were 158.8 million (95% CI, 152.1-165.4 million) patient visits among White individuals (85.6% of visits among White or Black individuals) and 26.7 million (95% CI, 24.6-28.7 million) patient visits among Black individuals (14.4%). Visits for Black individuals accounted for between 14.4% and 17.4% of visits of the period examined, and increases in telemedicine visits were similar among White individuals and Black individuals, with telemedicine

Downloaded From: https://jamanetwork.com/ by a University of Manitoba User on 06/09/2021

Table 1. Trends in Primary Care by Visit Type, 2018-2020[a]

Variable	No., in thousands (%)									
	2018				2019				2020	
	Q1	Q2	Q3	Q4	Q1	Q2	Q3	Q4	Q1	Q2
Total visits, No. (95% CI)	124 919 (119 697-130 141)	122 402 (117 286-127 518)	124 115 (118 927-129 303)	125 854 (120 593-131 115)	127 298 (121 786-132 810)	130 297 (124 655-135 939)	128 225 (122 673-133 777)	123 180 (117 846-128 514)	117 944 (112 648-123 240)	99 326 (94 866-103 786)
Office-based	116 614 (93.35)	114 249 (93.34)	115 780 (93.28)	117 219 (93.14)	117 741 (92.49)	121 344 (93.13)	117 936 (91.98)	114 176 (92.69)	105 911 (89.80)	58 668 (59.07)
Hospital-based	4126 (3.30)	3786 (3.09)	4189 (3.38)	4376 (3.48)	4949 (3.89)	4736 (3.63)	4951 (3.86)	4366 (3.54)	4229 (3.59)	3353 (3.38)
Telemedicine	1398 (1.12)	1194 (0.98)	1109 (0.89)	1363 (1.08)	1355 (1.06)	1611 (1.24)	1406 (1.10)	1369 (1.11)	4794 (4.07)	35 044 (35.28)
Other[b]	2780 (2.23)	3172 (2.59)	3037 (2.45)	2897 (2.30)	3253 (2.56)	2606 (2.00)	3933 (3.07)	3267 (2.65)	3009 (2.55)	2262 (2.28)

Abbreviation: Q, quarter.

[a] Source: IQVIA National Disease and Therapeutic Index, 2018-2020.[19]

[b] Other includes home, nursing home, and unspecified sites of care.

Downloaded From: https://jamanetwork.com/ by a University of Manitoba User on 06/09/2021

Table 2. Primary Care Office-Based and Telemedicine Visits by Patient Race, 2018-2020[a]

Variable	No., in thousands (%)[b]								
	Both White and Black individuals			White individuals			Black individuals		
	2018 (Q1/Q2)	2019 (Q1/Q2)	2020 (Q1/Q2)	2018 (Q1/Q2)	2019 (Q1/Q2)	2020 (Q1/Q2)	2018 (Q1/Q2)	2019 (Q1/Q2)	2020 (Q1/Q2)
All visits, No. (95% CI)	185 457 (177 705-193 209)	189 595 (181 386-197 804)	161 063 (153 831-168 295)	158 784 (152 147-165 421)	160 210 (153 273-167 147)	133 042 (127 068-139 016)	26 672 (24 648-28 696)	29 385 (27 075-31 695)	28 021 (25 737-30 305)
All visits, %	NA	NA	NA	85.6	84.5	82.6	14.4	15.5	17.4
Office-based	183 255 (98.8)	187 065 (98.7)	129 658 (80.5)	156 795 (98.7)	157 874 (98.5)	107 389 (80.7)	26 460 (99.2)	29 191 (99.3)	22 269 (79.5)
Telemedicine	2201 (1.2)	2530 (1.3)	31 405 (19.5)	1989 (1.3)	2336 (1.5)	25 653 (19.3)	212 (0.8)	194 (0.7)	5752 (20.5)

Abbreviations: NA, not applicable; Q, quarter.

[a] Source: IQVIA National Disease and Therapeutic Index, 2018-2020.[19]

[b] Values represent cumulative visits for each period.

Downloaded From: https://jamanetwork.com/ by a University of Manitoba User on 06/09/2021

visits accounting for 19.3% of 2020 treatment visits among White individuals and 20.5% of those of Black individuals.

eTable 2 in the Supplement presents the distribution of office-based and telemedicine visits stratified by patient age, sex, and insurance type (commercial, Medicaid, or other). Whereas individuals aged 19 to 35 years and aged 36 to 55 years accounted for 12.4% and 19.8% of office-based visits, respectively, in Q1/Q2 of 2020, they accounted for 17.8% and 26.1% of telemedicine visits, respectively, during this period, indicating substantial adoption of telemedicine compared with their younger or older counterparts (15.6% of telemedicine visits were individuals aged <19 years and 15.2% and 25.3% of visits were individuals aged 56-65 years and ≥66 years, respectively). Commercially insured visits accounted for an average of 60.3% of office-based visits for Q1 and Q2 of 2020 and an average of 57.3% of telemedicine visits for Q1 and Q2 of 2020.

Geographic Variation in Telemedicine Use

Table 3 and the **Figure** depict 2020 primary care office-based and telemedicine visits in the US stratified by geographic region. For example, during the first 2 quarters of 2020, there were 39.6 million (95% CI, 36.8-42.4 million) visits in the Pacific region (Washington, Oregon, and California). Of these visits, 10.6 million (95% CI, 9.2-12.1 million) (26.8%) were telemedicine encounters. The proportion of visits delivered by telemedicine varied from a low of 15.1% in the East North Central region (Wisconsin, Michigan, Illinois, Indiana, and Ohio) to a high of 26.8% in the Pacific region. Table 3 also presents the burden of the pandemic across these regions, with a case fatality rate that ranged from 19.90 to 124.91 per 100 000 individuals. There was no association between the use of telemedicine and the pandemic burden across geographic regions (r = 0.004; P = .99).

Features of Primary Care Office-Based and Telemedicine Visits

Table 4 and eTable 3 in the Supplement characterize the content of primary care visits over time, showing reductions in care assessment. For example, blood pressure was assessed in an estimated 88.7 million (95% CI, 84.6-92.7 million) Q2 visits in 2018/2019, 75.9 million (95% CI, 72.0-79.7 million) visits in Q1 of 2020, and 44.2 million (95% CI, 41.1-47.4 million) visits in Q2 of 2020, reflecting a 50.1% decrease (44.4 million visits) from Q2 2018-2019 levels. The absolute number of cholesterol assessments decreased 36.9% over the same period (10.2 million visits), while reductions in the absolute number of office-based and telemedicine visits with the initiation (26.0%) or continuation (8.9%) of new medicines were also noted.

One reason for this finding was that assessment of blood pressure and cholesterol was statistically significantly less common among telemedicine than among office-based visits. For example, of 58.7 million (95% CI, 55.3-62.1 million) Q2 2020 office-based visits, 69.7% had a blood pressure recorded compared with 9.6% of telemedicine visits during the same time period

Table 3. Primary Care Office-Based and Telemedicine Visits by Geographic Region and COVID-19 Burden, First 2 Quarters of 2020[a]

Geographic region	COVID-19 death rate (per 100 000 individuals)	Visits, No. in thousands (95% CI)		Telemedicine % of total
		Office-based	Telemedicine	
Pacific	19.90	29 000 (26 637-31 364)	10 631 (9158-12 104)	26.8
East South Central	24.23	9033 (7718-10 348)	1928 (1386-2470)	17.6
West South Central	25.30	14 792 (12 742-16 842)	2908 (2215-3601)	16.4
West North Central	21.11	8807 (7525-10 089)	1711 (1230-2192)	16.3
Mountain	27.62	9959 (8579-11 339)	2394 (1722-3066)	19.4
South Atlantic	29.65	36 238 (33 676-38 800)	8146 (6892-9400)	18.4
East North Central	44.97	27 402 (25 169-29 635)	4856 (3929-5783)	15.1
New England and Mid-Atlantic	124.91	29 349 (26 957-31 741)	7263 (6074-8452)	19.8
All regions	45.24	164 579 (157 189-171 969)	39 838 (37 021-42 655)	19.5

Abbreviation: COVID-19, coronavirus disease 2019.

[a] Source: IQVIA National Disease and Therapeutic Index, 2018-2020.[19]

Downloaded From: https://jamanetwork.com/ by a University of Manitoba User on 06/09/2021

($P < .001$). Cholesterol was also less commonly assessed during telemedicine than during office-based visits (13.5% vs 21.6% of Q2 2020 visits; $P < .001$). New prescription medications were ordered similar proportions of Q2 2020 telemedicine and office-based visits (39.3% vs 44.9%), but absolute numbers across these visit types decreased from 54.1 million (95% CI, 50.8-57.5 million) in Q2 2018/2019 to 51.8 million (95% CI, 48.5-55.1 million) in Q1 of 2020 and 40.1 million (95% CI, 37.2-42.9 million) in Q2 of 2020, representing a 26.0% decrease from Q2 2018-2019 volume.

Discussion

While the COVID-19 pandemic has impacted health care delivery in many ways, little is known regarding how the volume, site, and content of primary care in the US has changed. We used a nationally representative audit of outpatient care to characterize primary care delivery in the US

Figure. Geographic Variation in COVID-19 Burden and Telemedicine Adoption in the First 2 Quarters of 2020

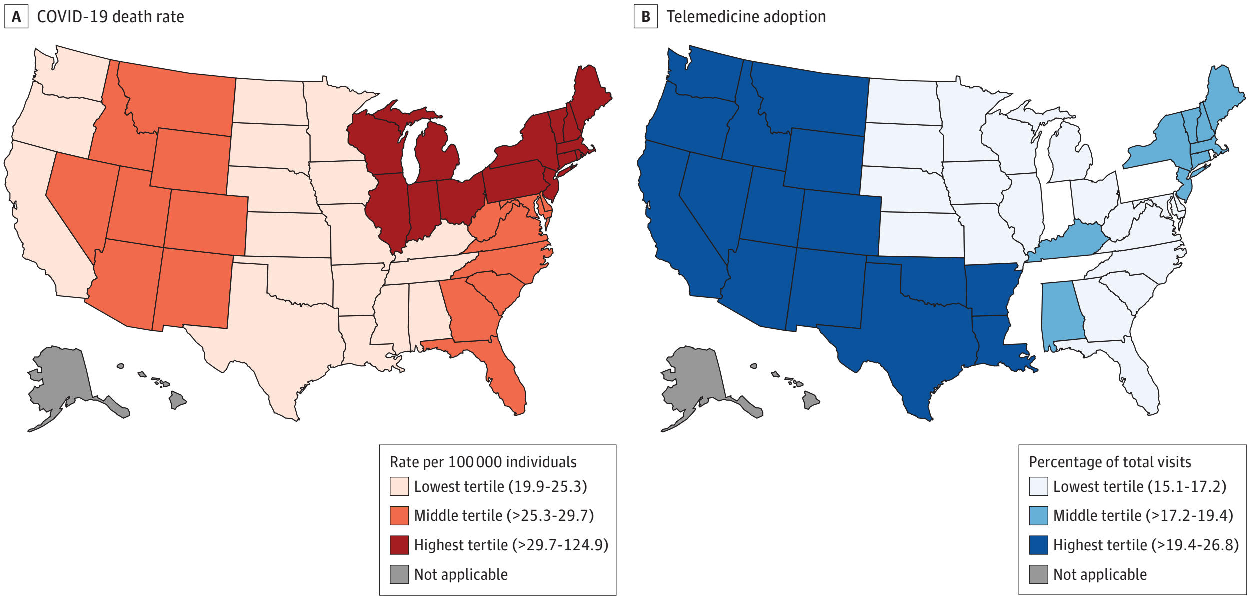

COVID-19 indicates coronavirus disease 2019.

Table 4. Content of Primary Care Office-Based and Telemedicine Visits, 2018-2020[a]

Variable	No., in thousands (%) 2018-2019 (Q2)	2020 (Q1)	2020 (Q2)	% Change (2020 Q2 vs 2018-2019 Q2)[b]
Total visits, No. (95% CI)	119 199 (114 038-124 360)	110 705 (105 734-115 676)	93 712 (89 270-98 154)	−21.4
Blood pressure recorded	88 675 (74.4)	75 852 (68.5)	44 229 (47.2)	−50.1
Cholesterol assessed	27 617 (23.2)	22 803 (20.6)	17 413 (18.5)	−36.9
New medicines initiated	54 142 (45.4)	51 773 (46.8)	40 079 (42.8)	−26.0
Medicines continued	38 024 (31.9)	35 541 (32.1)	34 621 (36.9)	−8.9
New treatment visits				
Hypertension	3414 (2.9)	2714 (2.5)	2078 (2.2)	−39.1
Diabetes	1408 (1.2)	1226 (1.1)	1177 (1.3)	−16.4
High cholesterol	1274 (1.1)	1326 (1.2)	926 (1.0)	−27.3
Asthma	1266 (1.1)	1146 (1.0)	635 (0.7)	−49.8
Depression	193 (0.2)	157 (0.1)	149 (0.2)	−22.8
Insomnia	396 (0.3)	437 (0.4)	299 (0.3)	−24.5

Abbreviation: Q, quarter.

[a] Values represent average second quarter visit volume (2018-2019) and quarterly visit volume (2020 Q1 and 2020 Q2).

[b] Percentage change depicts a comparison of the second quarter of 2020 (2020 Q2) with average volume of 2018 Q2 and 2019 Q2 combined.

Downloaded From: https://jamanetwork.com/ by a University of Manitoba User on 06/09/2021

between 2018 and Q2 of 2020. The pandemic has been associated with a more than 25% decrease in primary care volume, which has been offset in part by increases in the delivery of telemedicine, which accounted for 35.28% of encounters during the second quarter of 2020. Despite the increased use of telemedicine, its uptake has varied across the continental US and has not been correlated, at a regional level, with COVID-19 burden. Overall, the pandemic has been associated with marked reductions in the primary care assessment of cardiovascular risk factors such as blood pressure and cholesterol levels, owing to decreased total visit volume and less frequent assessment during telemedicine visits than during office-based visits. These findings are notable because little is known about the association between primary care delivery and the COVID-19 pandemic and because the pandemic has generated interest in telemedicine as a means to safely deliver primary care.

Our analysis was based on an assessment at a single point, and the degree to which the COVID-19 pandemic may be associated with permanent increases in the use of telemedicine remains to be seen. Historically, limited reimbursement, interstate licensure requirements, and patient and clinician factors have slowed the uptake and adoption of telemedicine.[12,23] In response to the pandemic, US federal and state agencies and other stakeholders have modified policies and procedures, such as the Centers for Medicare & Medicaid Services provision of telehealth waivers for providers,[24] to allow greater use of telemedicine to support remote clinical encounters.[25,26] We did not find a correlation between regional COVID-19 burden and telemedicine adoption, suggesting that other factors may account for regional differences in the uptake of this mode of health care. In addition, whether the federal and state rules and regulations that have been modified will be made permanent and whether the current embrace of telemedicine by patients and clinicians will endure remain unknown.[27]

If substantial primary care volume continues to be delivered using telemedicine, a focus on the content and quality of such encounters is inevitable.[28] Despite findings in a systematic review of 86 articles demonstrating the feasibility and acceptance of telemedicine for use in primary care, to our knowledge, relatively few rigorous comparisons of clinical outcomes in office-based vs telemedicine encounters have been performed.[29] Our finding that such visits were less likely to include blood pressure or cholesterol assessments underscores the limitation of telemedicine, at least in its current form, for an important component of primary care prevention and chronic disease management.

Middle-aged individuals and those who were commercially insured were more likely to adopt telemedicine during the pandemic than their counterparts with other or no insurance. This difference may be due in part to the perceived elective or deferrable nature of visits among children[30] and greater familiarity with telemedicine technology among middle-aged than among older adults.[31] We did not find substantial differences in telemedicine use by payer type, and, contrary to our expectations and evidence of a digital divide,[32] we did not find evidence of a racial disparity in telemedicine use when examining the frequency of telemedicine encounters as a proportion of a patient visits among Black versus White individuals.

Limitations

This study has limitations. First, both the COVID-19 pandemic and health system response continue to evolve, and our analyses reflect the provision of care at a single point in time. Second, as with any outpatient audit, the data that we used were subject to measurement error, although prior analyses have yielded estimates comparable to those from the National Ambulatory Medical Care Survey. Third, factors such as patient race are complex multidimensional constructs, and our assessment of race in this context provides a limited window through which to understand how telemedicine adoption may vary across different populations.[33,34] Fourth, our data did not allow us to examine more granular geographic associations between COVID-19 burden and telemedicine adoption. Last, we considered the assessment of 2 important cardiovascular risk factors, but many other dimensions of primary care might also be compared between office-based and telemedicine encounters, and we

Downloaded From: https://jamanetwork.com/ by a University of Manitoba User on 06/09/2021

did not attempt to assess the association between primary care encounter type and overall quality of care.

Conclusions

More than 4 months after the US Department of Health and Human Services declared a public health emergency, widespread economic and social changes in the US during the COVID-19 pandemic are still occurring. The pandemic has been associated with substantial decreases in primary care delivery, despite large increases in the use of telemedicine, which accounted for fewer than 2% of primary care visits during 2019 yet more than 35% of visits during Q2 of 2020. Evaluations of cardiovascular risk factors such as blood pressure and cholesterol have decreased, owing to fewer total visits and less frequent assessment during telemedicine encounters. Thus, the COVID-19 pandemic has been associated with changes in the structure of primary care, with the content of telemedicine visits differing from that of office-based encounters.

ARTICLE INFORMATION

Accepted for Publication: August 6, 2020.

Published: October 2, 2020. doi:10.1001/jamanetworkopen.2020.21476

Open Access: This is an open access article distributed under the terms of the CC-BY License. © 2020 Alexander GC et al. *JAMA Network Open*.

Corresponding Author: G. Caleb Alexander, MD, MS, Department of Epidemiology, Johns Hopkins Bloomberg School of Public Health, 615 N Wolfe St, W6035, Baltimore, MD 21205 (galexan9@jhmi.edu).

Author Affiliations: Center for Drug Safety and Effectiveness, Johns Hopkins Bloomberg School of Public Health, Baltimore, Maryland (Alexander, Tajanlangit, Heyward); Department of Epidemiology, Johns Hopkins Bloomberg School of Public Health, Baltimore, Maryland (Alexander, Heyward); Division of General Internal Medicine, Johns Hopkins Medicine, Baltimore, Maryland (Alexander); Student, Johns Hopkins University, Baltimore, Maryland (Tajanlangit); Monument Analytics, Baltimore, Maryland (Mansour); Department of Pharmacy Systems, Outcomes, and Policy, University of Illinois at Chicago, Chicago (Qato); Stanford Prevention Research Center, Stanford University, Palo Alto, California (Stafford).

Author Contributions: Dr Alexander had full access to all of the data in the study and takes responsibility for the integrity of the data and the accuracy of the data analysis.

Concept and design: Alexander, Heyward, Mansour, Stafford.

Acquisition, analysis, or interpretation of data: All authors.

Drafting of the manuscript: Alexander, Tajanlangit, Heyward, Stafford.

Critical revision of the manuscript for important intellectual content: Alexander, Heyward, Mansour, Qato, Stafford.

Statistical analysis: Tajanlangit, Mansour, Stafford.

Administrative, technical, or material support: Alexander, Heyward.

Supervision: Alexander, Heyward, Stafford.

Conflict of Interest Disclosures: Dr Alexander reported serving as past chair of the US Food and Drug Administration's Peripheral and Central Nervous System Advisory Committee; serving as a paid advisor to IQVIA; that he is a cofounding principal and equity holder in Monument Analytics, a health care consultancy whose clients include the life sciences industry as well as plaintiffs in opioid litigation; and that he is a member of OptumRx's National P&T Committee. This arrangement has been reviewed and approved by Johns Hopkins University in accordance with its conflict of interest policies. Dr Stafford reported serving as an unpaid advisor to IQVIA and receiving personal fees from the states of California, Washington, and Alaska outside the submitted work. No other disclosures were reported.

Disclaimer: The statements, findings, conclusions, views, and opinions contained and expressed in this article are based in part on data obtained under license from the following IQVIA Incorporated information services: IQVIA National Disease and Therapeutic Index (2018-2020), IQVIA Incorporated. All Rights Reserved. The statements, findings, conclusions, views, and opinions contained and expressed herein are not necessarily those of IQVIA Incorporated or any of its affiliated or subsidiary entities.

Downloaded From: https://jamanetwork.com/ by a University of Manitoba User on 06/09/2021

REFERENCES

1. Nicola M, Alsafi Z, Sohrabi C, et al. The socio-economic implications of the coronavirus pandemic (COVID-19): A review. *Int J Surg*. 2020;78:185-193. doi:10.1016/j.ijsu.2020.04.018

2. Cutler D. How will COVID-19 affect the health care economy? *JAMA Health Forum*. April 9, 2020. Accessed June 2, 2020. https://jamanetwork.com/channels/health-forum/fullarticle/2764547

3. Mehta HB, Ehrhardt S, Moore TJ, Segal JB, Alexander GC. Characteristics of registered clinical trials assessing treatments for COVID-19: a cross-sectional analysis. *BMJ Open*. 2020;10(6):e039978. doi:10.1136/bmjopen-2020-039978

4. Mehrotra A, Chernew M, Linetsky D, Hatch H, Cutler D. The impact of the COVID-19 pandemic on outpatient visits: a rebound emerges. The Commonwealth Fund: To the Point blog. May 19, 2020. Accessed May 28, 2020. https://www.commonwealthfund.org/publications/2020/apr/impact-covid-19-outpatient-visits

5. Coombs B. Telehealth visits are booming as doctors and patients embrace distancing amid the coronavirus crisis. CNBC website. April 4, 2020. Accessed May 28, 2020. https://www.cnbc.com/2020/04/03/telehealth-visits-could-top-1-billion-in-2020-amid-the-coronavirus-crisis.html

6. Mehrotra A, Chernew M, Linetsky D, Hatch H, Cutler D. The impact of the COVID-19 pandemic on outpatient visits: practices are adapting to the new normal. The Commonwealth Fund website. June 2020. Accessed July 27, 2020. https://www.commonwealthfund.org/publications/2020/jun/impact-covid-19-pandemic-outpatient-visits-practices-adapting-new-normal

7. US Dept of Justice, Drug Enforcement Administration. COVID-19 information page. Accessed March 18, 2020. http://www.deadiversion.usdoj.gov/coronavirus.html

8. US Dept of Health and Human Services. Telehealth: delivering care safely during COVID-19. Accessed June 2, 2020. https://www.hhs.gov/coronavirus/telehealth/index.html

9. Yang YT, Weintraub E, Haffajee RL. Telemedicine's role in addressing the opioid epidemic. *Mayo Clin Proc*. 2018;93(9):1177-1180. doi:10.1016/j.mayocp.2018.07.001

10. Federation of State Medical Boards. US States and territories modifying requirements for telehealth in response to COVID-19. May 26, 2020. Accessed June 2, 2020. https://www.fsmb.org/siteassets/advocacy/pdf/states-waiving-licensure-requirements-for-telehealth-in-response-to-covid-19.pdf

11. Scott Kruse C, Karem P, Shifflett K, Vegi L, Ravi K, Brooks M. Evaluating barriers to adopting telemedicine worldwide: A systematic review. *J Telemed Telecare*. 2018;24(1):4-12. doi:10.1177/1357633X16674087

12. Weinstein RS, Lopez AM, Joseph BA, et al. Telemedicine, telehealth, and mobile health applications that work: opportunities and barriers. *Am J Med*. 2014;127(3):183-187. doi:10.1016/j.amjmed.2013.09.032

13. Galewitz P. Telemedicine surges, fueled by coronavirus fears and shift in payment rules. Kaiser Health News. Published March 27, 2020. Accessed April 10, 2020. https://khn.org/news/telemedicine-surges-fueled-by-coronavirus-fears-and-shift-in-payment-rules/

14. Alexander GC, Gallagher SA, Mascola A, Moloney RM, Stafford RS. Increasing off-label use of antipsychotic medications in the United States, 1995-2008. *Pharmacoepidemiol Drug Saf*. 2011;20(2):177-184. doi:10.1002/pds.2082

15. Dorsey ER, Rabbani A, Gallagher SA, Conti RM, Alexander GC. Impact of FDA black box advisory on antipsychotic medication use. *Arch Intern Med*. 2010;170(1):96-103. doi:10.1001/archinternmed.2009.456

16. Higashi A, Zhu S, Stafford RS, Alexander GC. National trends in ambulatory asthma treatment, 1997-2009. *J Gen Intern Med*. 2011;26(12):1465-1470. doi:10.1007/s11606-011-1796-4

17. Stafford RS, Radley DC. The underutilization of cardiac medications of proven benefit, 1990 to 2002. *J Am Coll Cardiol*. 2003;41(1):56-61. doi:10.1016/S0735-1097(02)02670-0

18. Zell ER, McCaig LF, Kupronis BA, Besser RE, Schuchat A. A comparison of the National Disease and Therapeutic Index and the National Ambulatory Medical Care Survey to evaluate antibiotic usage. In: Proceedings of the survey research methods section, American Statistical Association. American Statistical Association. Accessed June 20, 2011. http://www.amstat.org/sections/srms/Proceedings/papers/2000_143.pdf

19. IQVIA. *National disease and therapeutic index*. IQVIA; 2020.

20. von Elm E, Altman DG, Egger M, Pocock SJ, Gøtzsche PC, Vandenbroucke JP; STROBE Initiative. The Strengthening the Reporting of Observational Studies in Epidemiology (STROBE) statement: guidelines for reporting observational studies. *J Clin Epidemiol*. 2008;61(4):344-349. doi:10.1016/j.jclinepi.2007.11.008

21. COVID-19 Dashboard by the Center for Systems Science and Engineering. Johns Hopkins University website. Accessed June 3, 2020. https://coronavirus.jhu.edu/map.html

22. World Population Review website. Accessed June 3, 2020. https://worldpopulationreview.com/states/

Downloaded From: https://jamanetwork.com/ by a University of Manitoba User on 06/09/2021

23. Ellimoottil C, An L, Moyer M, Sossong S, Hollander JE. Challenges and opportunities faced by large health systems implementing telehealth. *Health Aff (Millwood)*. 2018;37(12):1955-1959. doi:10.1377/hlthaff.2018.05099

24. Centers for Medicare & Medicaid Services. COVID-19 emergency declaration: blanket waivers for health care providers. Published June 25, 2020. Accessed July 28, 2020. https://www.cms.gov/files/document/summary-covid-19-emergency-declaration-waivers.pdf

25. American Medical Association. COVID-19 state policy guidance on telemedicine. Advocacy Resource Center webpage. Accessed June 3, 2020. https://www.ama-assn.org/system/files/2020-04/covid-19-state-policy-guidance-on-telemedicine.pdf

26. Alexander GC, Stoller KB, Haffajee RL, Saloner B. An epidemic in the midst of a pandemic: opioid use disorder and COVID-19. *Ann Intern Med*. 2020;173(1):57-58. doi:10.7326/M20-1141

27. Cutler DM, Nikpay S, Huckman RS. The business of medicine in the era of COVID-19. *JAMA*. 2020;323(20):2003-2004. doi:10.1001/jama.2020.7242

28. National Committee for Quality Assurance. COVID-driven telehealth surge triggers changes to quality measures: revisions reinforce the use of telehealth during the pandemic and after. Accessed June 4, 2020. https://www.ncqa.org/programs/data-and-information-technology/telehealth/covid-driven-telehealth-surge-triggers-changes-to-quality-measures/

29. Bashshur RL, Howell JD, Krupinski EA, Harms KM, Bashshur N, Doarn CR. The empirical foundations of telemedicine interventions in primary care. *Telemed J E Health*. 2016;22(5):342-375. doi:10.1089/tmj.2016.0045

30. Bramer CA, Kimmins LM, Swanson R, et al. Decline in child vaccination coverage during the COVID-19 pandemic: Michigan Care Improvement Registry, May 2016-May 2020. *MMWR Morb Mortal Wkly Rep*. 2020;69(20):630-631. doi:10.15585/mmwr.mm6920e1

31. American Well. Telehealth Index: 2019 Consumer Survey. Accessed August 19, 2020. https://static.americanwell.com/app/uploads/2019/07/American-Well-Telehealth-Index-2019-Consumer-Survey- eBook2.pdf

32. Lorence DP, Park H, Fox S. Racial disparities in health information access: resilience of the Digital Divide. *J Med Syst*. 2006;30(4):241-249. doi:10.1007/s10916-005-9003-y

33. LaVeist TA. Beyond dummy variables and sample selection: what health services researchers ought to know about race as a variable. *Health Serv Res*. 1994;29(1):1-16.

34. López L, Green AR, Tan-McGrory A, King R, Betancourt JR. Bridging the digital divide in health care: the role of health information technology in addressing racial and ethnic disparities. *Jt Comm J Qual Patient Saf*. 2011;37(10):437-445. doi:10.1016/S1553-7250(11)37055-9

SUPPLEMENT.

eTable 1. States Comprising IQVIA Subnational Regions in the National Disease and Therapeutic Index
eTable 2. Characteristics of Primary Care Office-based and Telemedicine Visits, 2018-2020 (in thousands)
eTable 3. Content of Primary Care Office-based and Telemedicine Visits, 2018-2020 (in thousands)
eFigure. Trends in Primary Care Stratified by Visit Type, 2018-2020

Downloaded From: https://jamanetwork.com/ by a University of Manitoba User on 06/09/2021
